# Genetic architecture, demographic history, and genomic differentiation of *Populus davidiana* revealed by whole‐genome resequencing

**DOI:** 10.1111/eva.13046

**Published:** 2020-07-15

**Authors:** Zhe Hou, Ang Li, Jianguo Zhang

**Affiliations:** ^1^ State Key Laboratory of Tree Genetics and Breeding Key Laboratory of Silviculture of the State Forestry Administration Research Institute of Forestry Chinese Academy of Forestry Beijing China; ^2^ Key Laboratory of Southwest China Wildlife Resources Conservation (Ministry of Education) China West Normal University Nanchong China; ^3^ Collaborative Innovation Center of Sustainable Nanjing Forestry University Nanjing China

**Keywords:** demographic history, genetic architecture, heterogeneous genomic differentiation, *Populus davidiana*, whole‐genome sequencing

## Abstract

Forest trees are an excellent resource from which to understand population differentiation and heterogeneous genome variation patterns due to the majority of forest trees being distributed widely and able to adapt to different climates and environments. *Populus davidiana* is among the most geographically widespread and ecologically important tree species in China. Whole‐genome resequencing data of 75 individual examples of *P. davidiana* throughout China were conducted, finding that all examples from different regions were clearly divided into either Northeast (N), Central (C), and South (S) populations. The ancestors of *P. davidiana* diverged into Northern group, comprising both N and C and Southern populations approximately 792,548 years ago. This time point of differentiation suggests that divergence of *P. davidiana* populations might have been triggered by the mid‐Pleistocene transition. The three populations experienced considerable periods of bottleneck following divergence, with population expansion beginning around 5,000 years ago after the end of the last glacial maximum. We found N to be the center of origin of *P. davidiana* in China. The migration route of *P. davidiana* in China was from N to S. Although the majority of the regions of genomic differentiation between N and S populations can be explained by neutral processes, a number of tested outlier regions were also found to have been significantly influenced by natural selection. Our results highlight that linked selection and rates of recombination were important factors in genomic differentiation between the N and S populations. Finally, we identified a substantial number of functional genes related to climate change during population differentiation and adaptive evolution.

## 1 INTRODUCTION

Understanding how genomes vary during population differentiation and how diverse evolutionary forces drive differentiation of the entire genome has received considerable attention in evolutionary biology research over recent years (Nachman & Payseur, 2012; Noor & Bennett, 2009; Nosil & Feder, 2012; Seehausen et al., 2014; Strasburg et al., 2012). In accordance with strict neutral theory, the mechanisms of genetic differentiation are the result of changing allele frequencies due to genetic drift and novel mutations (Hellmann et al., 2005). Demographic factors can trigger differentiation throughout the genome deviating from strict neutrality through a change in the effective population size such as population expansion or bottlenecks (Li & Durbin, 2011). Demographic fluctuations and genetic drift cause variation throughout the genome (Luikart, England, Tallmon, Jordan, & Taberlet, 2003). Nevertheless, Darwinian or natural selection affects only genes that provide important functional information. For example, both positive and purifying selection can cause genetic variation in reproductive isolation or ecological specialization loci that influence the fitness and respective phenotypes of an organism (Via, 2009). Recombination and mutation rates that affect important functional architecture of the entire genome are also essential evolutionary factors that determine the heterogeneity of genomic divergence (Nachman & Payseur, 2012; Noor & Bennett, 2009). In general, a combination of evolutionary factors affects the patterns of overall genomic variation during the process of population differentiation, such as demographic fluctuations, genetic drift, mutation, recombination rates, genetic hitchhiking, background selection, and migration, all performing important roles to shape the heterogeneity of genomic divergence (Wang, Street, Scofield, & Ingvarsson, 2016). Unraveling how the different evolutionary factors contribute to genomic differentiation, especially in parapatry, is important but challenging research.

With the development of high‐throughput sequencing technology, a growing quantity of genome‐wide data are becoming available and intense research activity has resulted in the discovery of substantial patterns of genetic variation and population divergence among multiple related species with considerably increased accuracy (Ellegren et al., 2012; Feulner et al., 2015; Turner, Hahn, & Nuzhdin, 2005). A universal interpretation of genetic differentiation from the overall genome suggests different levels of gene flow. A number of sites associated with reproductive isolation usually have higher levels of genetic differentiation, also commonly referred to as "genomic islands," whereas lower levels of variation are often observed in other sites across the genome due to gene flow (Nosil, Funk, & Ortizbarrientos, 2009). However, other studies have indicated that highly differentiated regions in the genome are incidental rather than directly related to ecological speciation. The authors have argued that highly differentiated regions occur because linked selection (positive and purifying selection) substantially reduces genetic diversity by removing neutral polymorphism and increases genome divergency, especially in regions with low rates of recombination (Cruickshank & Hahn, 2014). Furthermore, long‐term balancing selection increases variability within a population resulting in low genetic differentiation between species (Charlesworth, Charlesworth, & Morgan, 1995). It is now apparent that the different forms of natural selection (positive, purifying, and balancing selection) alone are enough to shape the different patterns of genomic differentiation (Turner et al., 2005). Finally, genomic divergence deviating from the strict neutrality model can also be shaped by neutral forces, such as demographic fluctuations, mutation, and stochastic genetic drift (Campagna, Gronau, Silveira, Siepel, & Lovette, 2015; Nosil et al., 2009). In order to unravel the patterns of heterogeneous genomic divergence, detailed information about the population differentiation process and accurate model calculations is required because those neutral forces described above are not mutually exclusive (Nosil & Feder, 2012).

The majority of forest trees are distributed widely and can adapt to variations in climate and the environment without any anthropogenic influence, and harbor a wealth of genetic variation. Therefore, they are an excellent resource for understanding population differentiation and genome variation patterns (Neale & Kremer, 2011). *Populus davidiana* Dode (Salicaceae) is among the most geographically widespread (across latitudes) and ecologically important tree species in China, which has persisted largely in an undomesticated state that is highly resistant to different environmental stresses (Hou et al., 2018). *Populus davidiana* has high ecological and economic value with high levels of intraspecific genetic diversity. A recent study of *P. davidiana* based on six nuclear and three chloroplast loci suggests that three distinct groups, from the Northeast, Central, and Southwest China, exist and that a refugium might have existed in Northeast China during the last glacial maximum (LGM; Hou et al., 2018).

*Populus davidiana* has wide geographical distribution, high intraspecific polymorphism, and adaptability to different environments, combined with a relatively small genome size. Consequently, *P. davidiana* represents an excellent model for understanding how different evolutionary forces have sculpted the variation patterns in the genome during the process of population differentiation and ecological speciation. In the present research study, next‐generation sequencing (NGS) was used to analyze 75 *P. davidiana* trees to explore population structure, estimate population divergence time points, identify the historical demographic processes, and infer the overall patterns of genomic divergence. Finally, multiple genes related to adaptation to local environments were identified in *P. davidiana*. This study provides insights into the evolutionary history and genetic diversity of the species, in addition to describing examples of the mechanisms by which a species can adapt to regions with variations in climate. At the same time, the study also provides the important reference value for understanding the mechanism of the formation of the geographical distribution patterns of other plant populations in China.

## 2 MATERIALS AND METHODS

### 2.1 Sample collection

**Figure 1:**
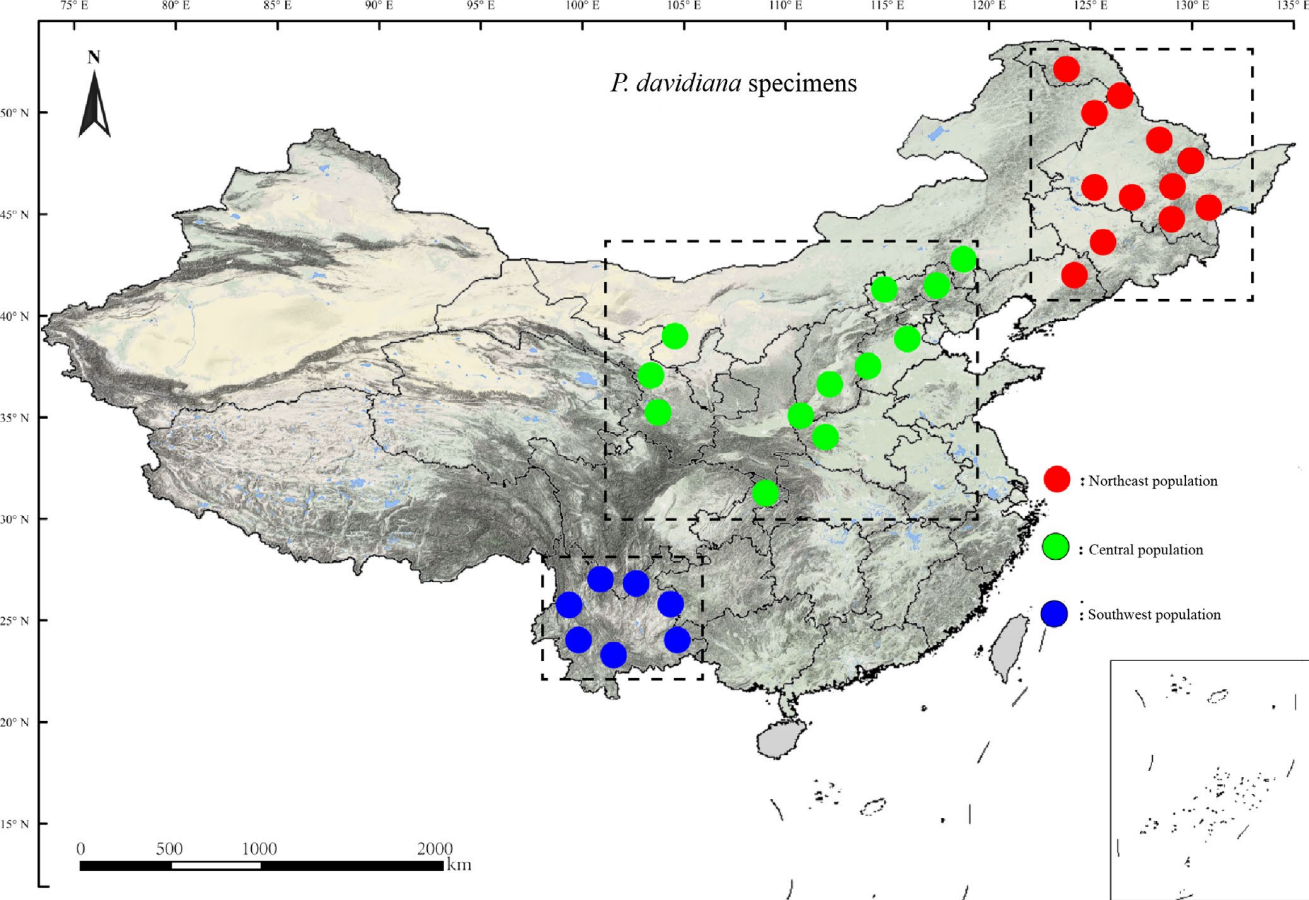
Geographical distribution of 75 *Populus davidiana*

A total of 30 individual trees from the Northeast, 21 from the Central, and 24 from the Southwest of China were collected from 32 natural populations of *P. davidiana* (Figure 1; Table S1). The genomic DNA from all 75 specimens was extracted from ~25 mg fresh leaves in accordance with a modified CTAB method (Doyle, 1987). The extracted DNA was quantified and quality‐checked at A260/A280 nm (NanoDrop, Thermo Fisher Scientific) prior to sequencing. The DNA sample was normalized to a standard concentration (50 ng/µl) in preparation for sequencing/library prep.

### 2.2 Sequencing, quality control, and data processing

A paired‐end sequencing library was prepared for every *P. davidiana* specimen and sequencing performed from high‐quality DNA based on the standard Illumina HiSeq 2000 platform protocol with an expected target coverage of 30×. The raw sequence data reported in this paper have been submitted to the Genome Sequence Archive (Wang et al., 2017) at the BIG Data Center, Beijing Institute of Genomics (BIG), Chinese Academy of Sciences, under accession number CRA001592, and are publicly accessible at http://bigd.big.ac.cn/gsa. Adapter sequences of the raw data were removed using Trimmomatic software (Lohse et al., 2012) prior to read alignment. Strict quality control was conducted with elimination of a base from either the beginning or end of each read if the base quality value was less than 20. Furthermore, reads were completely filtered out if the length of the sequence was less than 36 bases after trimming. After quality control, the BWA‐MEM algorithm (Heng Li et al., 2009) was used with parameters: “‐t 8 ‐k 32 ‐M –R” to map all clean data to the *P. trichocarpa* reference genome, version 3 (Tuskan, 2006). SAMtools (Heng Li et al., 2009) was used to sort the resulting reads after mapping and RealignerTargetCreator and IndelRealigner applications (Depristo et al., 2011) used to conduct local realignment for correcting mismatched bases due to insertions and/or deletions. Duplicated reads were removed using MarkDuplicates available in the Picard application (http://broadinstitute.github.io/picard). After investigating the empirical distribution, we removed sites showing extremely low (<100 reads across all samples per species) or high (>1,200 reads across all samples per species) read coverage. In addition, reads or sites that included >20 mapping quality scores of zero within the whole sample were discarded. Finally, only top‐quality reads were retained for downstream analyses.

### 2.3 SNP and genotype calling

We employed two complementary approaches for SNP and genotype calling. ANGSD v0.928 (Korneliussen, Albrechtsen, & Nielsen, 2014) is a classic software package for the analysis of genome sequencing data, which was employed to estimate the site frequency spectrum (SFS), but not to call genotypes. Low‐quality data were filtered out, with reads that had a mapping quality <30 and bases with a quality score <20 not considered with the parameters ‐minQ 20 ‐minMapQ 30. The SAMTools genotype likelihood model (Li et al., 2009) with the parameter ‐doSaf implemented to estimate SFS probability for calculating all population genetic statistics. HaplotypeCaller and GenotypeGVCFs modules in GATK v3.7.1 (Depristo et al., 2011) were used to perform accurate genotype and SNP calls. In order to improve the reliability and accuracy of SNP and genotype calling, four strict filtering steps were employed to decrease false positives and error rate in the data: (a) All low‐quality SNPs that failed the previous filtering step were completely deleted; (b) SNPs >2 alleles were discarded from all sites; (c) biallelic SNPs <5 bp were deleted and SNPs >5 bp retained from each indel; and (d) GQ (genotype quality) scores of less than 10 suggested missing genotype data, so all SNPs with >2 instances of missing genotypes were discarded.

### 2.4 Population structure

NGSadmix software (Skotte, Korneliussen, & Albrechtsen, 2013) was utilized to infer population genetic structure in *P. davidiana*, and sites with less than 10% of their data missing were used, the number of coancestry clusters (K) ranging from 1 to 6. Principal component analysis (PCA) was performed using PCAngsd software (http://www.popgen.dk/software/index.php/PCAngsd). TreeBest software was employed (http://treesoft.sourceforge.net/treebest.shtml) to construct neighbor‐joining (NJ) phylogenetic trees, with *Populus tremula* used as an out‐group. We downloaded the data of *P. tremula* from the Short Read Archive (SRA) at NCBI, and the accession numbers are SRR2744682, SRR2745906, SRR2745908, and SRR2745909. In addition, all sample covariance matrix was estimated using PCAngsd software and a relevance clustering thermal map was plotted using Omicshare Tools software (http://www.omicshare.com/tools/Home/Soft/getsoft/type/index).

### 2.5 Demographic history

A coalescent simulation‐based method was employed to infer demographic histories and the mode of differentiation for the three different *P. davidiana* populations determined using Fastsimcoal 2.6.1 software (Excoffier, Dupanloup, Huerta‐Sánchez, Sousa, & Foll, 2013). Allele frequencies in the 75 samples were calculated using the realSFS module in ngsTools software so as to construct the required two‐dimensional joint site frequency spectrum (2D‐SFS), which was estimated with 100,000 coalescent simulations in each model. Twenty different models were formulated to simulate the past population histories of *P. davidiana* that differed in terms of: (a) AsymmetricMigration without population expansion; (b) NoMigration without population expansion; (c) AsymmetricMigration with population expansion; (d) complex model, including a bottleneck in N; and (e) complex model, including a bottleneck in S (Figure S3, Table S2). Every model ran independently 50 times and performed 15–45 conditional maximization algorithm cycles to obtain a global maximum likelihood that could be used to evaluate the results. The run having the greatest likelihood was selected from 50 independent runs for comparison between different models. AIC (Akaike information criterion) and Akaike's weight were used as criteria for judging. The model exhibiting the largest Akaike's weight was used as the best (Excoffier et al., 2013). In the calculation, we assumed an annual mutation rate at each locus of the *Populus* species of 2.5 × 10^–9^, with 15 years as a generation (Koch, Haubold, & Mitchell‐Olds, 2000). Among 100 parametric bootstraps, parameter confidence intervals were selected from the best model from 50 independent runs.

The effective population size (Ne) was then evaluated over a historical time frame through utilization of multiple sequential Markovian coalescent (MSMC) analyses (Schiffels & Durbin, 2014) in three different *P. davidiana* populations. Prior to performing the calculation, all segregating sites within each population were phased and imputed using Beagle software (Browning, Zhou, & Browning, 2018). Fifteen years was assumed as the generation time and an annual mutation rate at each locus of the *Populus* species of 2.5 × 10^–9^ (Tuskan, 2006) to convert the scaled time and effective population size to actual time and size.

### 2.6 Genome‐wide patterns of differentiation and detection in outlier windows

VCFtools software (Petr et al., 2011) was used to calculate *F*_ST_ and Tajima's D, using sliding windows with a nonoverlapping window size of 10 kbp. *F*_ST_ values were then sorted, the top 1% and negative end of Tajima's *D* values selected as a highly differentiated region using a selective sweep (Chen et al., 2018). We detected a poorly differentiated region with an *F*_ST_ value less than 0.15. We estimated two summary statistics, nucleotide diversity π and Tajima's *D*, from sample allele frequency likelihoods in ANGSD for all simulation replicates to test whether the simulated data matches the observed data. To assess whether any of the observed windows display *F*_ST_ values deviating significantly from neutral expectations, we determined the conditional probability (*p*‐value) of observing more extreme interspecific *F*_ST_ values among simulated data sets than among the observed data. The determination of significance was based on running 500,000 coalescent simulations of the most acceptable demographic null model. We then corrected for multiple testing by using the false discovery rate (FDR) adjustment, and only windows with FDR lower than 1% were considered as candidate regions affected by selection.

### 2.7 Population genetic analysis and molecular signatures of selection in outlier regions

To assess selection signals in both highly and poorly differentiated regions, multiple population genetic parameters of the two unions of outer regions were compared with the rest of the genome in the Northeast and South populations. Firstly, ANGSD was employed to estimate sample allele frequency probabilities between populations of the Northeast and South with a nonoverlapping window size of 10 kbp for calculating θπ, Fay and Wu (2000) and Fu and Li (1993). Secondly, VCFtools v0.1.12b was used to evaluate the linkage disequilibrium (LD) value over each 10 kbp window and to calculate the correlation coefficients (*r* ^2^) between SNPs with pairwise distances larger than 1 kbp (Danecek et al., 2011). FastEPRR software (Gao, Ming, Hu, & Li, 2016) was used to calculate recombination rates (ρ) over a window size of 10,000 bp. Finally, ngsStat software (Fumagalli, Vieira, Linderoth, & Nielsen, 2014) was used to evaluate four other genetic differentiation parameters, as follows: (a) Among all segregating sites, the fixed difference caused by derivation of the alleles fixed in the populations of the Northeast or South was calculated, with *P. tremula* used as the out‐group; (b) interspecific shared polymorphisms were calculated among all segregating sites; (c) the difference between two nucleotides (dxy) was calculated based on the posterior probability of the sample allele frequency at each locus over a window size of 10,000 bp; (d) the relative node depth (RND) was calculated by dividing the dxy of the Northeast and South populations by the dxy between the Northeast population and *P. tremula*. For all population genetic parameters, Wilcoxon ranked‐sum tests were used to examine the significance of differences between outlier regions and the remainder of the genome.

### 2.8 Genes under positive selection

We applied the Hudson–Kreitman–Aguadé (HKA) test (Hudson, Kreitman, & Aguadé, 1987) and the population branch statistic (PBS; Yi et al., 2010) to verify whether recent positive selection had acted specifically in the highly differentiated regions of N and S populations. We considered only coding regions and analyzed a total of 26,856 genes to identify genes under positive selection. For each gene, we recorded the number of polymorphic sites in one population (A) (such as the N population) and the number of fixed differences (the sites with *F*_ST_ > 0.95) between this population and both of the other two populations (B) (such as N and S populations). We then performed the HKA test by comparing the ratio of A/B to the genome‐wide average, computed as the sum of A and B values across all genes analyzed and testing the null hypothesis A(gene)/B(gene) = A(genome‐wide)/B(genome‐wide) using a Pearson's chi‐square test (Yi et al., 2010). In addition, we also calculated the population branch statistic (PBS). Finally, genes with a significant nominal *p*‐value (<.01) for the HKA test and a ranked PBS above the 95th percentile were considered as positively selected genes.

### 2.9 Gene ontology (GO) and KEGG pathways enrichment

GO enrichment analysis was utilized to examine which functional genes were overrepresented in highly differentiated regions, and agriGO’s Term Enrichment tool used (http://bioiSo.cau.edu.cn/agriGO/index.php) to perform a Fisher's exact test (Du, Zhou, Ling, Zhang, & Su, 2010). Multiple tests were performed using the Benjamini–Hochberg error detection rate to further correct the *p*‐value of the Fisher’s exact test (Bandelt, Forster, & Röhl, 1999). Significantly enriched GO terms with a *p*‐value less than .05 were identified. We employed the KOBAS system (Mao, Tao, & Wei, 2005) to analyze the KEGG pathways and the FDR method implemented to correct the various comparisons.

## 3 RESULTS

A total of 75 *P. davidiana* whole‐genome resequenced data were generated for downstream analysis. The genomes of *P. davidiana* and *P. trichocarpa* are highly conserved (Pakull, Groppe, Meyer, Markussen, & Fladung, 2009), such that more than 88.08% (Table S1) of all *P. davidiana* sequences can be mapped to the reference genome of *P. trichocarpa* (Tuskan, 2006) following a quality control process. The mean coverage of each site reached 32.7 in mapped reads of *P. davidiana* samples (Table S1). Two different but complementary methods were used to obtain reliable SNP and genotype data: (a) ANGSD software (Korneliussen et al., 2014) was used to produce high‐quality site‐frequency‐spectrum (SFS) data for estimating population genetic parameters without calling genotypes (Nielsen, 2005). (b) The HaplotypeCaller function in GATK software (Danecek et al., 2011) was used to call SNPs for evaluating other required accurate genotype calls. After filtration and strict quality control, a total of 5,863,539 high‐quality GATK SNP sites were obtained for additional analysis.

### 3.1 Population structure

**Figure 2:**
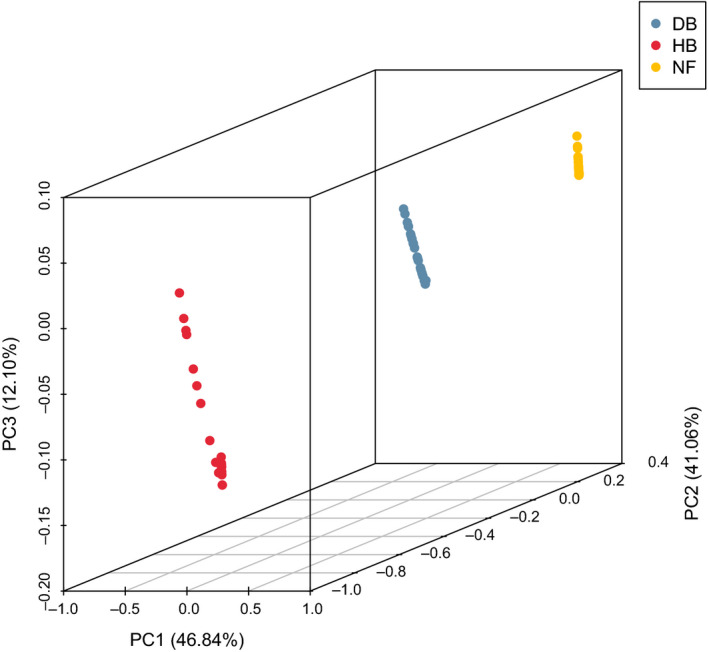
Principal component analysis (PCA) was performed using PCAngsd

NGSadmix was used to infer the genetic structure of *P. davidiana*. For *K* = 2, all 75 *P. davidiana* individuals were clearly divided into two groups: Northern and Southern populations. Further population sub‐structuring was observed in the Northern population when *K* = 3, where individuals from populations of the Northeast and Central regions of China clustered into two subgroups. When *K* = 4, no additional genetic structuring was observed (Figure S1). A neighbor‐joining tree was also constructed using *P. tremula* as an out‐group that further supported these patterns, with different geographical locations from the Northeast to the Southwest reflecting the grouping of populations (Figure S2). PCA also supported the results above, with the 75 *P. davidiana* trees from different regions clearly divided into populations from the Northeast (N), Central (C), and South (S). We found that the first three components explained 46.84%, 41.06%, and 12.10% of total genetic variance according to a Tracy–Widom test, respectively (Figure 2). The results of the relevance clustering thermal map further supported these patterns, finding also that N had the highest correlation with C, and N the lowest correlation with S (Figure S3). In terms of the number of polymorphisms, fixed differences accounted for 26% among the three populations, whereas shared polymorphism loci represented 36%, and private polymorphic loci in the N, C, and S populations accounted for 12%, 7%, and 9%, respectively (Figure S4).

### 3.2 Demographic histories

**Figure 3:**
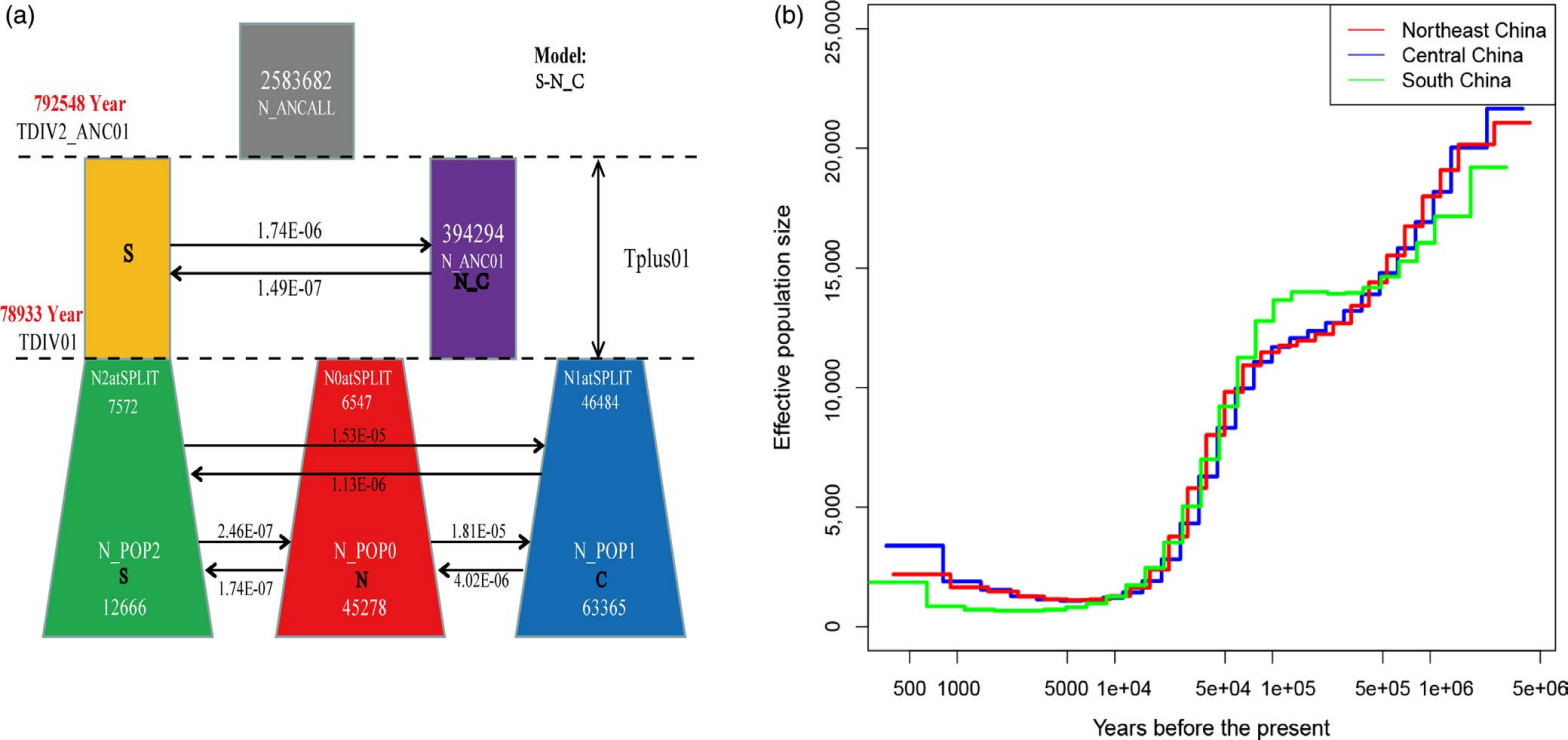
(a) Best‐fitting model inferred demographic histories and differentiation mode for three different *P. davidiana* populations implemented by fastsimcoal 2.6.1. (b) The effective population size (*Ne*) over historical time implementing by MSMC

A coalescent simulation‐based method was employed to infer demographic histories of *P. davidiana* using Fastsimcoal 2.6.1 software (Excoffier et al., 2013). The most appropriate model was one of complex isolation‐with‐migration. The ancestry of *P. davidiana* firstly diverged into Northern and Southern populations, after which the Northern ancestor differentiated into N and C populations (Figure 3a). A detailed effective population size, differentiation time point, and gene flow of *P. davidiana* are displayed in Table 1, which also presents the 95% confidence interval (CIs) for the related parameters. The ancestors of *P. davidiana* diverged into Northern and Southern populations approximately 792,548 years ago (bootstrap range [BR]: 780,000–800,000). The ancestry of the Northern population differentiated into N and C populations approximately 78,933 ago (bootstrap range [BR]: 77,865–79,025). The current effective population sizes (Ne) of N (N_e‐N_), C (N_e‐C_), and S (N_e‐S_) are 45,278 (BR: 43,688–46,671), 63,365 (BR: 62,321–65,105), and 12,666 (BR: 11,123–13,105), respectively. The effective population sizes of the three populations are all significantly lower than their common ancestor (N_e‐ANC_ = 2,573,682 [2,480,215–2,600,225]). The migration rate (m) is also clear among the three populations, the lowest generation migration rate (m) between N and S populations (1.74 × 10^–7^ and 2.46 × 10^–7^), which is not unexpected given the large geographical distance and disjunct distributions between the two populations.

**Table 1:** Demographic parameters and confidence interval of the best model

Parameters	Point estimation	95% CI^a^	
		Lower bound	Upper bound
N_e‐ANC_	2,573,682	2,480,215	2,600,225
N_e‐N_	45,278	43,688	46,671
N_e‐C_	63,365	62,321	65,105
N_e‐S_	12,666	11,123	13,105
m_N‐>C_	1.81 × 10^–5^	1.68 × 10^–5^	1.85 × 10^–5^
m_C‐>_ *_N_*	4.02 × 10^–6^	3.95 × 10^–6^	4.25 × 10^–6^
m_N‐>S_	1.74 × 10^–7^	1.68 × 10^–7^	1.86 × 10^–7^
m_S‐>N_	2.46 × 10^–7^	2.25 × 10^–7^	2.56 × 10^–7^
m_C‐>S_	1.13 × 10^–6^	1.05 × 10^–6^	1.25 × 10^–6^
m_S‐>C_	1.53 × 10^–5^	1.46 × 10^–5^	1.58 × 10^–5^
TDIV_North_South_	792,548	780,000	800,000
TDIV_N_C_	78,933	77,865	79,025

Note

Parameters are defined in Figure 7. N_e‐ANC_, N_e‐N_, N_e‐C,_ and N_e‐S_ indicate the effective population sizes of ancestral, N, C, and S populations, respectively, m_N‐>C_ indicates the per generation migration rate from N to C, m_C‐>N_ indicates the per generation migration rate from C to N, m_N‐>S_ indicates the per generation migration rate from N to S, m_S‐>N_ indicates the per generation migration rate from S to N, m_C‐>S_ indicates the per generation migration rate from C to S, m_S‐>C_ indicates the per generation migration rate from S to C, TDIV_North_South_ indicates the estimated divergence time between Northern and Southern populations, and TDIV_N_C_ indicates the estimated divergence time between N and C populations.

^a^ Parametric bootstrap estimates obtained by parameter estimation from 100 data sets simulated according to the overall maximum composite likelihood estimates shown in point estimation columns. Estimations were obtained from 100,000 simulations per likelihood.

The effective population size (*Ne*) over historical time was also evaluated using MSMC software (Schiffels & Durbin, 2014) in the three different *P. davidiana* populations. The recent effective population size will be more apparent when a greater number of samples and haplotypes have been analyzed (Schiffels & Durbin, 2014). Four individuals and eight haplotypes were used to infer changes in *N*_e_ for each population. Additional numbers were not used so as to limit computing cost. The three populations experienced considerably long periods of bottleneck following divergence, with population expansion beginning approximately 5,000 years ago after the end of the LGM (Figure 3b).

### 3.3 Genome differentiation and identification of outlier regions

**Figure 4:**
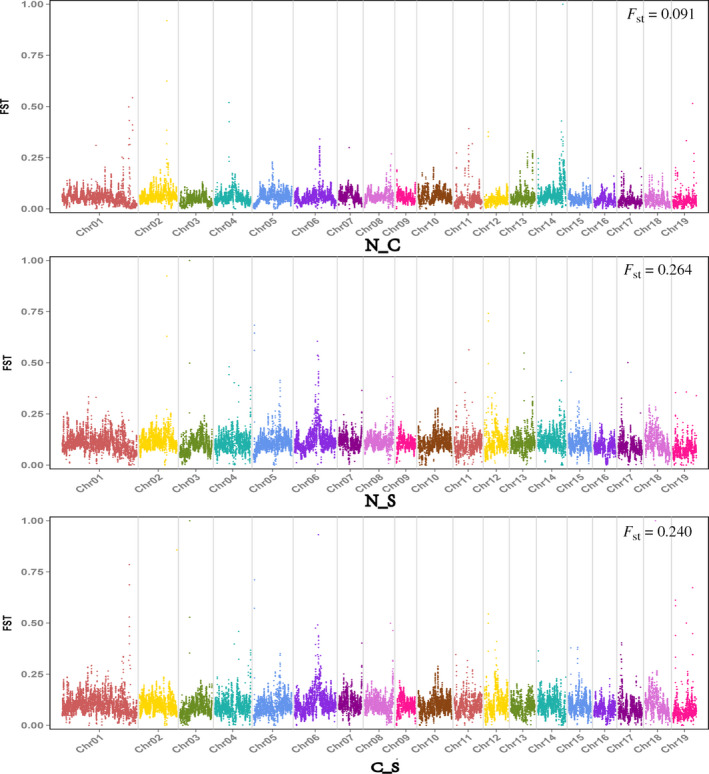
Pairwise *F*_ST_ between *P. davidiana* populations through each chromosome

**Figure 5:**
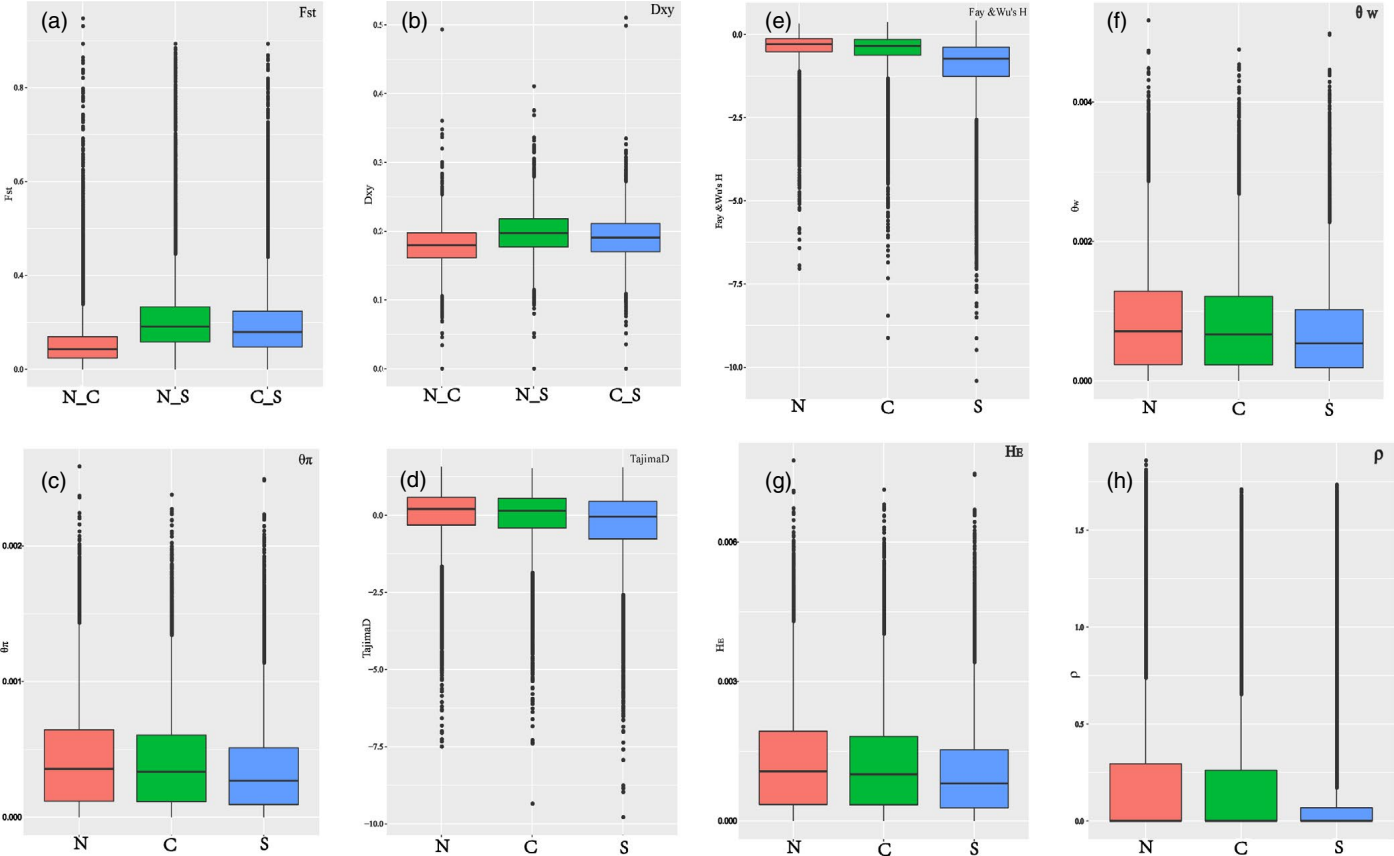
Population genetic analysis. (a) Pairwise *F*_ST_ between *P. davidiana* populations through the whole genome; (b) the difference between two nucleotides (dxy); (c) nucleotide diversity (θ_π_); (d) Tajima's D; (e) Fay & Wu's H; (f) Watterson's estimator (θ_W_); (g) gene diversity/heterozygosity (HE); (h) recombination rate (ρ)

The fixation index *F*_ST_ is a standard genetic differentiation parameter and therefore sensitive to any process that alters interspecific variation (Cruickshank & Hahn, 20142014). In the present study, the genetic differentiation coefficient *F*_ST_ was calculated for the three populations. We found that genetic differentiation was evident between the three populations, with highly differentiated regions randomly distributed in many different regions across the genome (Figure 4). From the overall level of the genome, clear genetic differentiation levels were found among the three populations. The *F*_ST_ values between N and S, S and C, and N and C were 0.264, 0.240, and 0.091, respectively (Figures 4 and 5a). The most apparent genetic differentiation was observed between the N and S populations. This may be because the geographical locations of the two populations are farthest apart. We also calculated dxy, total sequence differentiation between the populations, an absolute criterion for evaluation of interspecific differentiation. Sequence differentiation was also evident among the three populations, with dxy values between N and S, S and C, and N and C found to be 0.203, 0.198, and 0.189, respectively (Figure 5b). The result is consistent with the observation of *F*_ST_.

*F*_ST_ was calculated between N and S using 10,000 bp windows to investigate the genetic differentiation patterns between N and S populations across the genome. N and S populations exhibited the greatest degree of differentiation. *F*_ST_ was calculated between N and S using 10,000 bp windows to investigate the genetic differentiation patterns across the genome. The top 1% of *F*_ST_ values and the negative end of Tajima's *D* values were selected as highly differentiated regions with a selective sweep (Chen et al., 2018). We detected a poorly differentiated region with an *F*_ST_ value of less than 0.15.

We identified 674 and 262 outlier windows exhibiting significantly (false discovery rate < 0.01) high and low interspecific *F*_ST_, randomly distributed throughout the genome, and these outlier windows possibly affected by natural selection.

### 3.4 Population genetic analysis

The genome contains a large number of inspectable neutral loci and evolutionary information. We also calculated many population genetic parameters in N, C, and S populations, including Watterson's estimator (θ_W_), Tajima's D, Fay & Wu's H, nucleotide diversity (π), recombination rate (ρ), gene diversity/heterozygosity (*H*_E_), and linkage disequilibrium (LD) (*r* ^2^) to infer whether the N population possessed the greatest genetic diversity and represented the origin of *P. davidiana* in China as previously suggested (Hou et al., 2018). This was also valuable as an important reference to ascertain whether the N population was the center of adaptability and diversification. Throughout the genome, we observed that the genetic diversity parameters *π* (Figure 5c), *θ*_W_ (Figure 5f), and *H*_E_ (Figure 5g) of the N population were highest, and the S population had the lowest genetic diversity. Tajima's D (Figure 5d) and Fay & Wu's H (Figure 5e) parameters of the N population were >0 and that of the S population <0. The recombination rate ρ of the N population was much higher than that of S population (Figure 5h).

The LD decay distance generally refers to the physical distance when the mean LD coefficient *r* ^2^ decays to half of the maximum value. The N, C, and S populations of *P. davidiana* exhibited different LD decay curves (Figure S8), suggesting that the demographic histories of the three populations were diverse. The LD pattern of the genome may be altered by population reduction or genetic differentiation. The N population possessed the smallest LD value and fastest decay rate, while the S population had the largest LD value and slowest decay rate (Figure S8). Moreover, the current effective population of the S was smallest, and we speculate that the evolutionary force generated by this small population size in the formation of LD is strongest.

### 3.5 Signatures of selection in outlier regions

**Figure 6:**
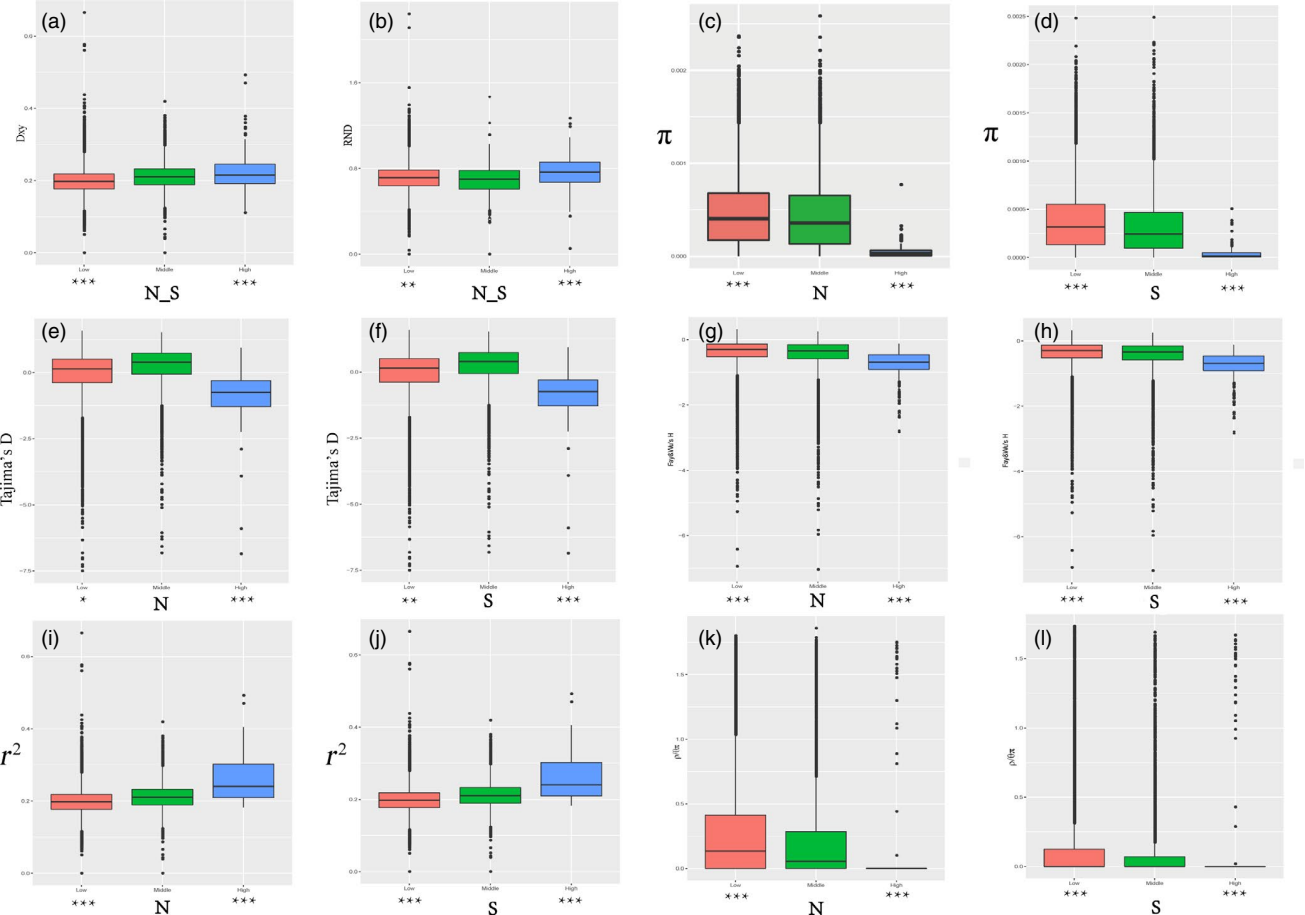
The outlier regions that have been tested to be significantly influenced by natural selection. (a) Comparisons of dxy among regions displaying significantly high (blue boxes) and low (red boxes) differentiation versus the genomic background (green boxes) between N and S populations; (b) comparisons of RND between N and S populations; (c) comparisons of nucleotide diversity π in N; (d) comparisons of nucleotide diversity π in S; (e) comparisons of Tajima's D in N; (f) comparisons of Tajima's D in S; (g) comparisons of Fay & Wu's H in N; (h) comparisons of Fay & Wu's H in S; (i) comparisons of *r* ^2^ in N; (j) comparisons of *r* ^2^ in S; (k) comparisons of recombination rate (ρ/θ_π_) in N; (l) comparisons of recombination rate (ρ) in S. Asterisks designate significant differences between outlier windows and the rest of genomic regions by Mann–Whitney *U* test (*p‐value < .05; **p‐value < 1e−4; ***p‐value < 2.2e−16)

**Figure 7:**
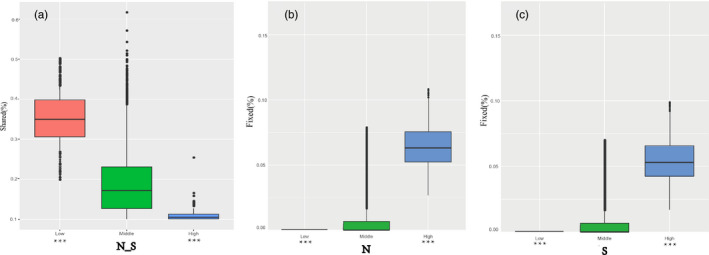
The proportion of interspecific shared polymorphisms (a) and the proportion of fixed differences caused by derived alleles fixed in either *N* (b) or S (c)

We compared multiple population genetic parameters of the two unions of outlier regions to the remaining parts of the genome in the N and S populations to assess selection signals in both highly differentiated and poorly differentiated regions. The dxy and RND values of the highly differentiated regions between the two populations showed significantly greater differentiation compared with regions of low differentiation. We also found that highly differentiated regions of the two populations had distinct positive selection characteristics (Nielsen, 2005). For example, the level of polymorphism (π) of both N and S populations were extremely low (Figure 6c,d). The more negative Tajima's D values revealed rare alleles that appeared frequently (Figure 6e,f), whereas the more negative Fay & Wu's H demonstrated derived alleles that appeared frequently (Figure 6g,h). A more apparent feature was the highly differentiated regions with stronger signals of linkage disequilibrium (LD) (Figure 6i.j; *p* < .001, Mann–Whitney *U* test). We also compared alleles fixed in the N or S populations and interspecific shared polymorphisms between the two populations. The results indicated that the proportion of interspecific shared polymorphisms in the highly differentiated regions was extremely low (Figure 7a) and the proportion of fixed differences significantly high in both the N and S populations (Figure 7b,c). To explore the genetic basis of this differentiation, we conducted HKA (Hudson–Kreitman–Aguadé) and PBS (population branch statistic) tests to identify genes under positive selection in the highly differentiated regions of each population. Among 26,856 genes analyzed, a total of 208 were identified under positive selection . This further suggests that positive selection was the principal evolutionary force driving the differentiation.

However, regions of low differentiation had long‐term balancing selection characteristics (Charlesworth et al., 1995). For example, the dxy and RND values of the regions that were poorly differentiated between the two populations exhibited less differentiation compared with regions exhibiting high differentiation (Figure 6a,b), the level of polymorphism (π) in both N and S populations being significantly high (Figure 6c,d). The higher Tajima's D and Fay & Wu's H parameters revealed intermediate‐frequency alleles that appeared frequently (Figure 6e‐h), with levels of LD that were lower than in the highly differentiated regions, possibly influenced by recombination (Lee, Yong, & Hyun, 2011). The proportion of interspecific shared polymorphisms in the poorly differentiated regions was higher (Figure 7a) and the proportion of fixed differences negligible in both the N and S populations (Figure 7b,c).

### 3.6 Effect of recombination rate on genome differentiation

Recombination rate is also an important factor affecting genome differentiation. FastEPRR software was used to calculate recombination rates (ρ = 4*N*_e_c) over a window size of 10,000 bp. Because ρ = 4*N*_e_c, a decrease in *N*_e_ in a region associated with selection reduces the local estimate of ρ. To eliminate this effect, we evaluated the effect of recombination rate on genomic differentiation by calculating ρ/θ_π_ in poorly and highly differentiated regions. In particular, we found a significant negative correlation between *F*_ST_ and the rate of recombination. The rate of recombination of the highly differentiated regions was extremely low, with a poorly differentiated region with a higher recombination rate (Figure 6k,l). These results indicate that recombination rate played an important role in the process of genomic differentiation in the N and S populations of *P. davidiana*.

### 3.7 Genes under selection

Annotation of the *P. trichocarpa* reference genome allows us to perform functional annotations on highly differentiated regions of *P. davidiana*. A total of 223 selected genes were identified in the present study. Gene ontology (GO) was used to analyze the differential enrichment of candidate genes, and we found that GO terms related to electron transport, apoptotic process, programmed cell death, and cell death were significantly overrepresented (Figure S6). The functional genes associated with electron transport were most highly enriched, with a total of 14 genes that were significantly overrepresented. After analysis, 11 genes related to photosynthesis were found, accounting for almost 80% (Table S3). KEGG pathway enrichment analysis using the KOBAS system also found that functional genes related to photosynthesis were significantly enriched (Figure S7). We also found 11 functional genes related to apoptosis and programmed cell death were also significantly enriched. It is worth noting that all 11 genes were related to plant disease resistance (Table S4). These results indicate that functional genes related to photosynthesis and plant disease resistance played an important role in the differentiation process of the N and S populations.

## 4 DISCUSSION

High‐throughput sequencing technology is a valuable tool for deciphering population structure and demographic history, enabling discovery of fine‐scale genetic variation and population divergence across the genome. We used whole‐genome resequencing to explore genetic architecture, demographic history, and genomic differentiation of *P. davidiana* in different populations throughout China. We found that *P. davidiana* was roughly divided into three groups according to their geographical distribution: a Southern (S) group, a Northeast (N) group, and a Central (C) group. We calculated *F*_ST_ and dxy values across the genome and found that there was clear genetic differentiation among the three populations. To comprehend how diverse evolutionary forces drove the differentiation and demographic history of the three different populations, we applied a whole‐genome resequencing approach based on a large number of unbiased SNPs distributed across the genome.

### 4.1 Demographic history of the three different populations

A coalescent simulation‐based method was employed to infer demographic histories of *P. davidiana* in Fastsimcoal 2.6.1 software (Excoffier et al., 2013). Our analyses indicated that the ancestors of *P. davidiana* diverged into Northern and Southern populations approximately 792,548 years ago (Figure 3a). The time point of differentiation is highly consistent with the mid‐Pleistocene transition (MPT). The MPT is widely recognized as an extraordinary transition of ice‐age cycles from 41‐ to 100‐kyr between 0.9 and 0.7 Ma (mega annum), largely reflecting the significant glacial–interglacial changes in high‐latitude ice volume (Hays, Imbrie, & Shackleton, 1976), global ocean temperature (Herbert, Peterson, Lawrence, & Liu, 2010), sea level (Rohling et al., 2014), and monsoonal climate (deMenocal, 1995).

The Northern population of *P. davidiana* differentiated into N and C populations approximately 78,933 years ago. The most recent glacial period occurred approximately 0.11–0.02 Ma, during which there were dramatic climatic oscillations. Such fluctuations and historical tectonic changes led to the differentiation and even extinction of the majority of plants in Northern China during the last glacial period (Cheng, Hwang, & Lin, 2005; Lu, Peng, Cheng, Hong, & Chiang, 2001; Shen et al., 2005; Zhang, Chiang, George, Liu, & Abbott, 2005). Quaternary climate fluctuations and regional uplift readily resulted in geographical isolation among different populations (Han et al., 2017), including the appearance of glacial refugia which fragmented the distribution of the species. A number of studies have shown that in the last glacial period, there were glacial refugia in Northern China, which provided protection for the survival of various species (Tian et al., 2009; Zeng, Wang, Liao, Wang, & Zhang, 2015). Our previous research also found evidence to support the existence of glacial refugia for *P. davidiana* in Northern and other geographical distributions of *P. davidiana,* and that the distribution range had also been fragmented (Du et al., 2015). Geographical isolation impeded gene flow between the populations (Hancock & Bergelson, 2011). In addition, due to the different selection pressures on different populations, the isolated populations gradually accumulated variation, resulting in differentiation between the different populations.

Therefore, Quaternary dramatic climate oscillations, historical tectonism, glacial refugia, extremely low temperatures, and geographical isolation impeded gene flow between different populations resulting in differentiation of *P. davidiana* in China. Population differentiation in other plant species due to historical Quaternary tectonism and climate oscillations in the middle Pleistocene have also been studied in China (Jin, Brown, & Liu, 2010; Qi et al., 2013; Via, 2009; Yang et al., 2009). MSMC indicated that the three populations experienced a considerable long‐term bottleneck after divergence, with population expansion beginning approximately 5,000 years ago after the end of the LGM (Figure 3b). This demographic is consistent with many other forest trees in Eurasia, the initiation of the population expansion in *P. davidiana* coincided with the end of the LGM (Hewitt, 2000, 2004).

### 4.2 Origin and evolution of *P. davidiana* in China

*Populus davidiana* is among the most geographically widespread (from Northeast to Southwest) and ecologically important tree species in China and has been shown to be clearly divided into N, C, and S populations. Therefore, it is of interest to study the origin and migration route of *P. davidiana*, because of the important reference value for understanding the mechanism of the formation of the geographical distribution patterns of other plant populations in China.

The genome contains a great deal of evolutionary information with inspectable neutral loci, and we evaluated several population genetic parameters to infer the origin and evolution of *P. davidiana* in China. Throughout the genome of the three populations, we found that the values of π (Figure 5c), θ_W_ (Figure 5f), and *H*_E_ (Figure 5g) in the N population were much higher than in the S population, clearly indicating that the N population possessed the highest genetic diversity and S the lowest. The minimal genetic diversity of the Tibetan barley species, qingke, suggests that Tibet is unlikely to be the center of origin or domestication for barley (Shancen et al., 2013). Similarly, we can infer that S was not the center of origin of *P. davidiana* in China. Tajima's D (Figure 5d) and Fay & Wu's H (Figure 5e) of the N population were greatest, indicating that intermediate‐frequency alleles appeared frequently (Wang et al., 2016). The recombination rate ρ of the N population was considerably higher than that of the S population (Figure 5h). Furthermore, the N population had the smallest LD value and fastest decay rate, while the S population had the largest LD value and slowest decay rate (Figure S8).

In summary, the N population exhibited the highest genetic diversity, greatest intermediate‐frequency alleles, highest recombination rate, lowest LD, and the fastest decay rate. All these factors indicate that N is the center of origin of *P. davidiana*. The migration route of *P. davidiana* in China was from N to S.

### 4.3 Heterogeneous genomic differentiation of N and S

Due to the large geographical distance and disjunct distributions between N and S populations, so stochastic genetic drift and loss of gene flow led to the accumulation of interspecific differentiation (Chen et al., 2018). We detected a large number of genomic differentiation regions between the two populations. Although the majority of these in the two populations can be explained by neutral processes (Strasburg et al., 2012), some outlier regions were tested that are significantly influenced by natural selection (Nielsen, 2005). The *F*_ST_ value would be expected to be high in those regions with a low recombination rate if natural selection was the principal evolutionary factor for genetic differentiation of the two populations (Noor & Bennett, 2009), because natural selection, such as selective sweeps, and background selection remove neutral variation, especially in areas with very low recombination rates (Begun et al., 2007). Accordingly, relative measures of divergence (*F*_ST_) and absolute divergence (dxy) will be higher, depending on intraspecific genetic diversity in areas with lower rates of recombination (Nachman & Payseur, 2012; Noor & Bennett, 2009). Consistent with the observations above, we found a significant negative correlation between *F*_ST_ and recombination rate (ρ) in both N (Figure 6k) and S (Figure 6l) populations (Keinan & Clark, 2012). As a consequence, our results highlight that linked selection and ρ were important factors of genomic differentiation between N and S populations (Cruickshank & Hahn, 2014; Turner et al., 2005).

The highly differentiated regions in the present study did not just cluster into large regions of the genome (Cruickshank & Hahn, 2014; Turner et al., 2005), but into narrow differentiation islands throughout the genome. The majority of the islands were located in regions with restricted recombination. Linked selection included positive selection (advantageous mutations) and purifying selection (deleterious mutations), which are also referred to as genetic hitchhiking and background selection (Cruickshank & Hahn, 2014; Noor & Bennett, 2009; Turner et al., 2005). Therefore, we evaluated numerous population genetic parameters to comprehend how genomic variation occurred during population differentiation and how diverse evolutionary forces drove the differentiation of the entire genome in N and S populations (Figure 6). We found that the highly differentiated regions of the two exhibited the characteristics of significant positive selection (Nielsen, 2005). For example, the level of polymorphism (π) in both N and S populations was extremely low (Figure 6c,d). The more negative Tajima's D revealed that rare alleles appeared frequently (Figure 6e,f), whereas the more negative Fay & Wu's H indicated that derived alleles appeared frequently (Figure 6g,h). A more apparent feature was that the highly differentiated regions exhibited stronger signals of linkage disequilibrium (Figure 6i,j) and higher dxy and RND values (Figure 6a,b) showing absolute intraspecific divergence. And a total of 208 genes were identified under positive selection in the highly differentiated regions. Our findings thus highlight significant effects of linked selection in generating the heterogeneous differentiation landscape we observe between the two populations. Under the process of linked selection, although genetic diversity was reduced, population differentiation increased. We found that genes related to electron transport, apoptotic process, programmed cell death, and cell death were significantly overrepresented within these regions (Figure S7). After analysis, 11 genes related to photosynthesis were found (Table S3) and 11 genes related to plant disease resistance (Table S4), suggesting that after differentiation of N and S populations, adaptive evolution of the two populations involved a large number of functional categories and genes (Wolf & Ellegren, 2017). However, since it is difficult to accurately estimate the variation in these highly differentiated regions exhibiting low genetic diversity, more caution is required in interpreting the functional characteristics of the overrepresented genes identified here. Therefore, more in‐depth research is required on these functional genes in order to clarify how widespread forest tree species respond to climate change during adaptive evolution.

In addition to the characteristics of positive selection being found in the highly differentiated regions, we also identified long‐term balancing selection in the poorly differentiated regions in both populations (Charlesworth et al., 1995). For example, absolute interspecific divergence (dxy and RND values) was lower than in the highly differentiated regions (Figure 6a,b). The genetic diversity (π) of both N and S populations was significantly high (Figure 6c,d). Higher Tajima's D and Fay & Wu's H values revealed that intermediate‐frequency alleles appeared frequently (Figure 6e–h), with levels of LD lower than the highly differentiated regions, which may have been influenced by recombination (Lee et al., 2011). The proportion of interspecific shared polymorphisms in the poorly differentiated regions was higher (Figure 7a) and the proportion of fixed differences negligible in both the N and S populations (Figure 7b,c).

## 5 CONCLUSIONS

*Populus davidiana* are distributed widely and ecologically important tree species in China and represents an excellent model for understanding how different evolutionary forces have sculpted the variation patterns in the genome during the process of population differentiation and ecological speciation. In the present study, we provide insights into population differentiation and the evolutionary history of a geographically widespread tree species in China, *P. davidiana*. The study indicated that all *P. davidiana* throughout China were clearly divided into N, C, and S populations. The ancestors of *P. davidiana* diverged into Northern and Southern populations around 792,548 years ago as a result of the MPT. The Northern population differentiated into N and C populations approximately 78,933 years ago. Population genetic analysis indicated that N represents the center of origin of *P. davidiana* in China. The migration route of *P. davidiana* in China was from N to S. Although the majority of regions of genomic differentiation between the two populations can be explained by neutral processes, some outlier regions have also been tested that are significantly influenced by natural selection. Our results highlight that linked selection and rates of recombination were important factors in genomic differentiation between the N and S populations. We identified multiple functional genes related to photosynthesis and plant disease resistance that played an important role in the differentiation process of the N and S populations. Our research highlights that more information needs to be integrated into future work when interpreting genomic variation during population differentiation. These include strict neutral theory, demographic fluctuations, genetic drift, geographical isolation, gene flow, sources of adaptation, positive selection (advantageous mutations), and purifying selection (deleterious mutations). These findings have implications for rethinking the evolutionary history of other temperate forest species in East Asia as well as the role of these evolutionary factors in maintaining high species diversity in this region.

## CONFLICT OF INTEREST

The authors declare that the research was conducted in the absence of any commercial or financial relationships that could be construed as a potential conflict of interest.

## AUTHOR CONTRIBUTIONS

Z.H. performed the experiments and wrote the study. J.G.Z. designed the research.

## Supporting information

Figure S1

Figure S2

Figure S3

Figure S4

Figure S5

Figure S6

Figure S7

Figure S8

Tables S1‐S4

## Data Availability

The raw sequence data reported in this paper have been submitted in the Genome Sequence Archive (Wang et al., 2017) in BIG Data Center, Beijing Institute of Genomics (BIG), Chinese Academy of Sciences, under accession number CRA001592, and are publicly accessible at http://bigd.big.ac.cn/gsa.
