# Supplementary figures and images for: Genetic architecture, demographic history, and genomic differentiation of Populus davidiana revealed by whole‐genome resequencing
Source: Evol Appl. 2020 Jul 15;13(10):2582–96. doi: 10.1111/eva.13046 (PMC7691461; doi:10.1111/eva.13046)

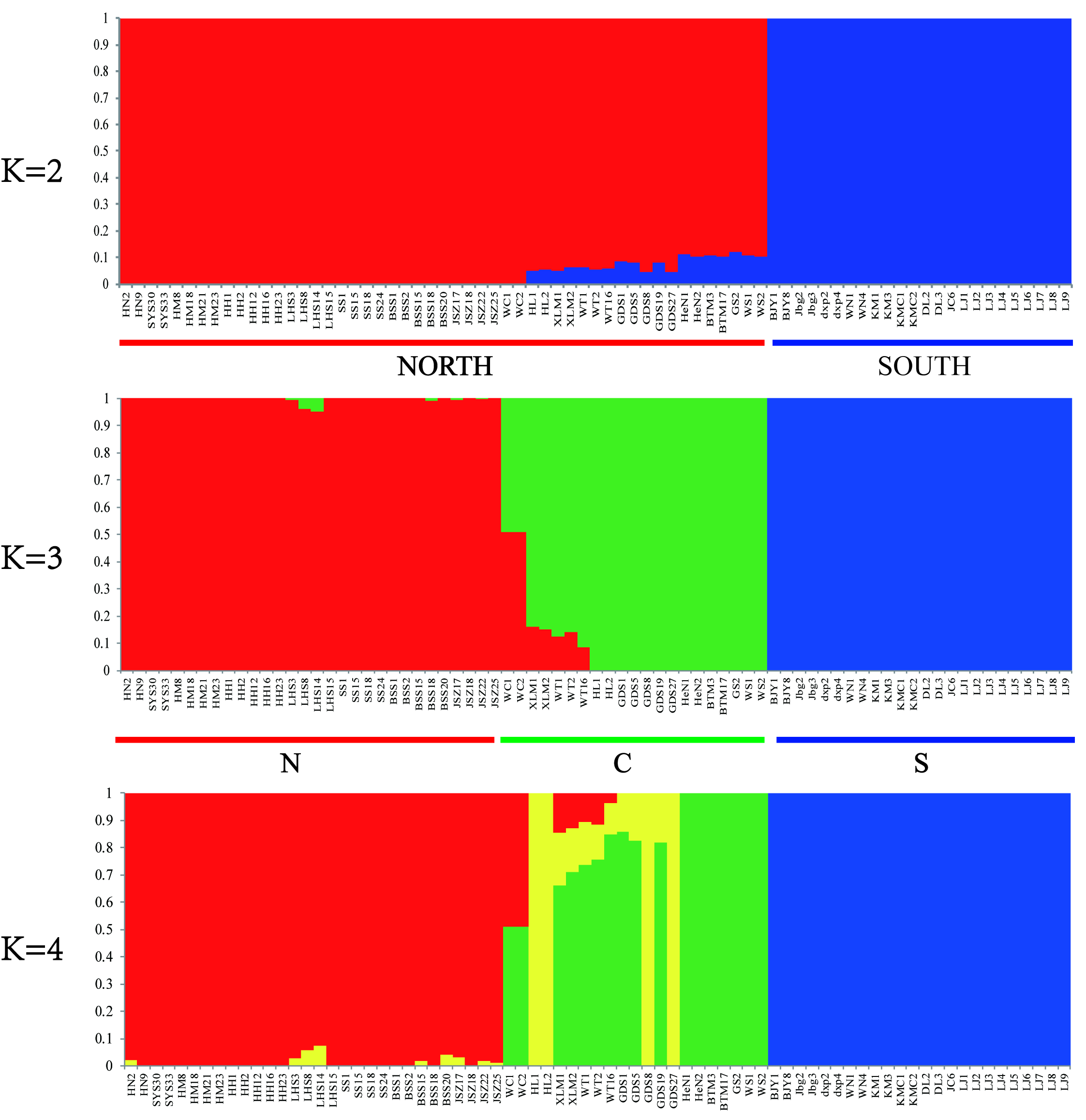

Supplement: Supplementary file 1 — Figure S1 [file EVA-13-2582-s001.tif]

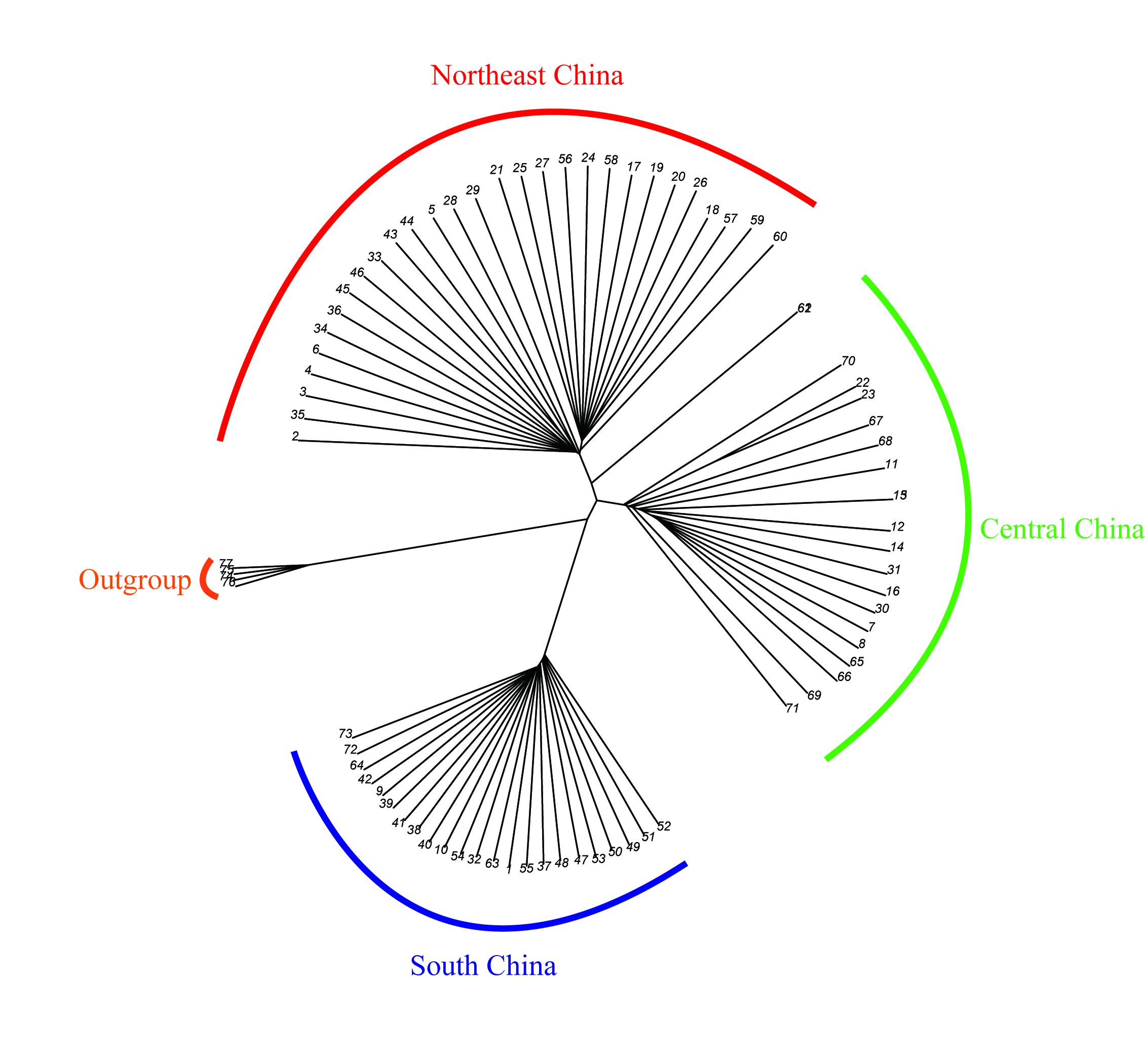

Supplement: Supplementary file 2 — Figure S2 [file EVA-13-2582-s002.tif]

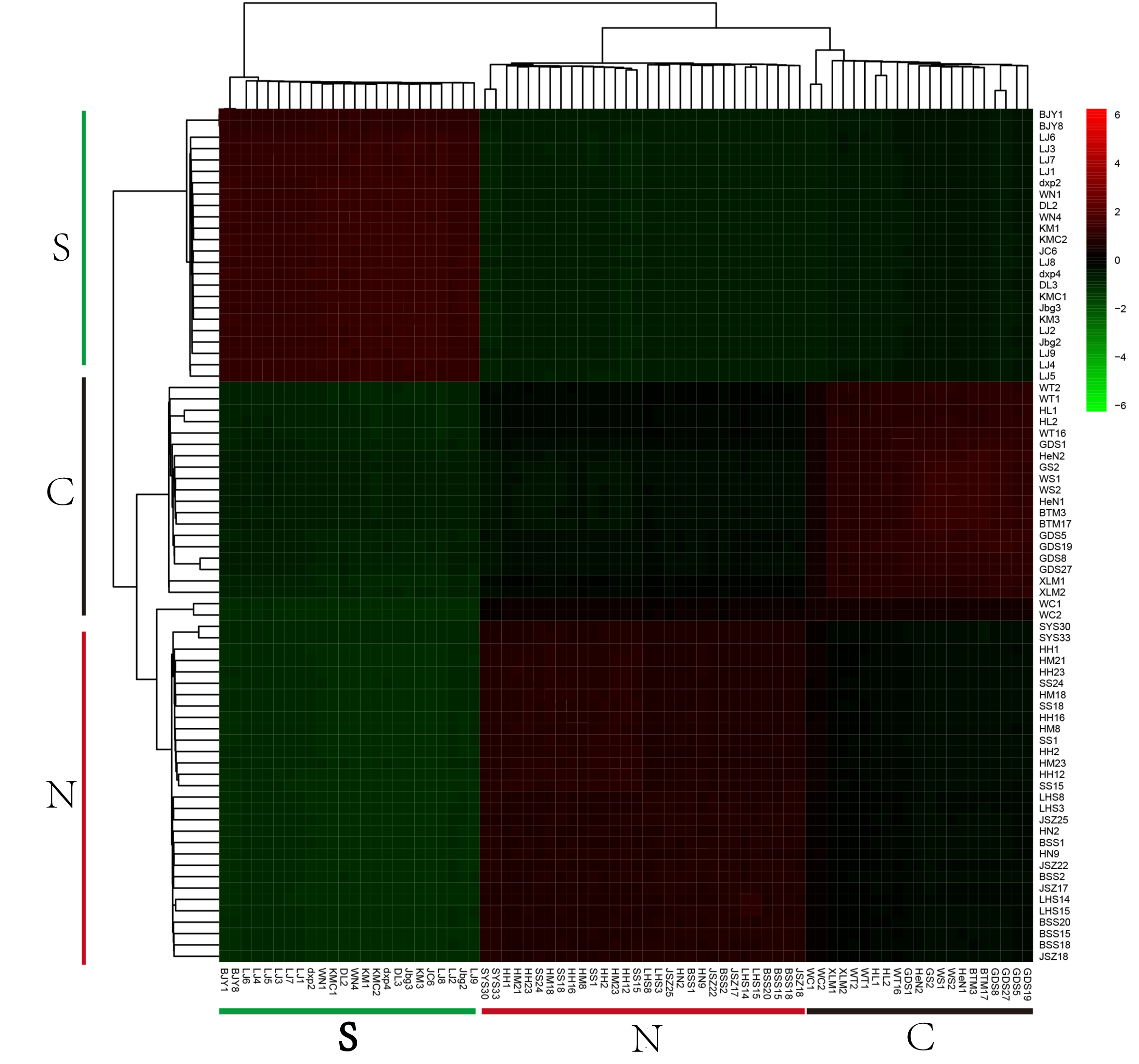

Supplement: Supplementary file 3 — Figure S3 [file EVA-13-2582-s003.tif]

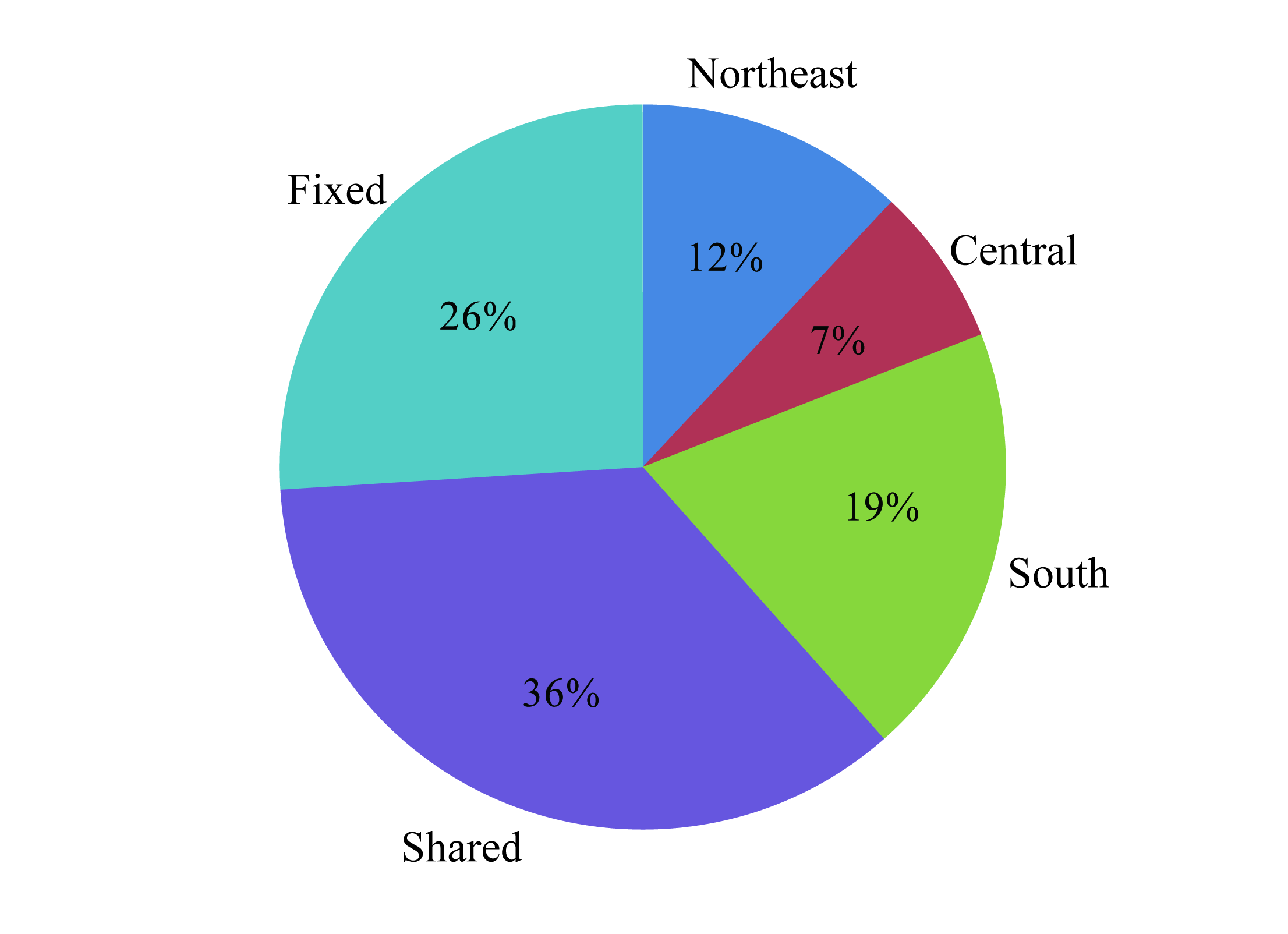

Supplement: Supplementary file 4 — Figure S4 [file EVA-13-2582-s004.tif]

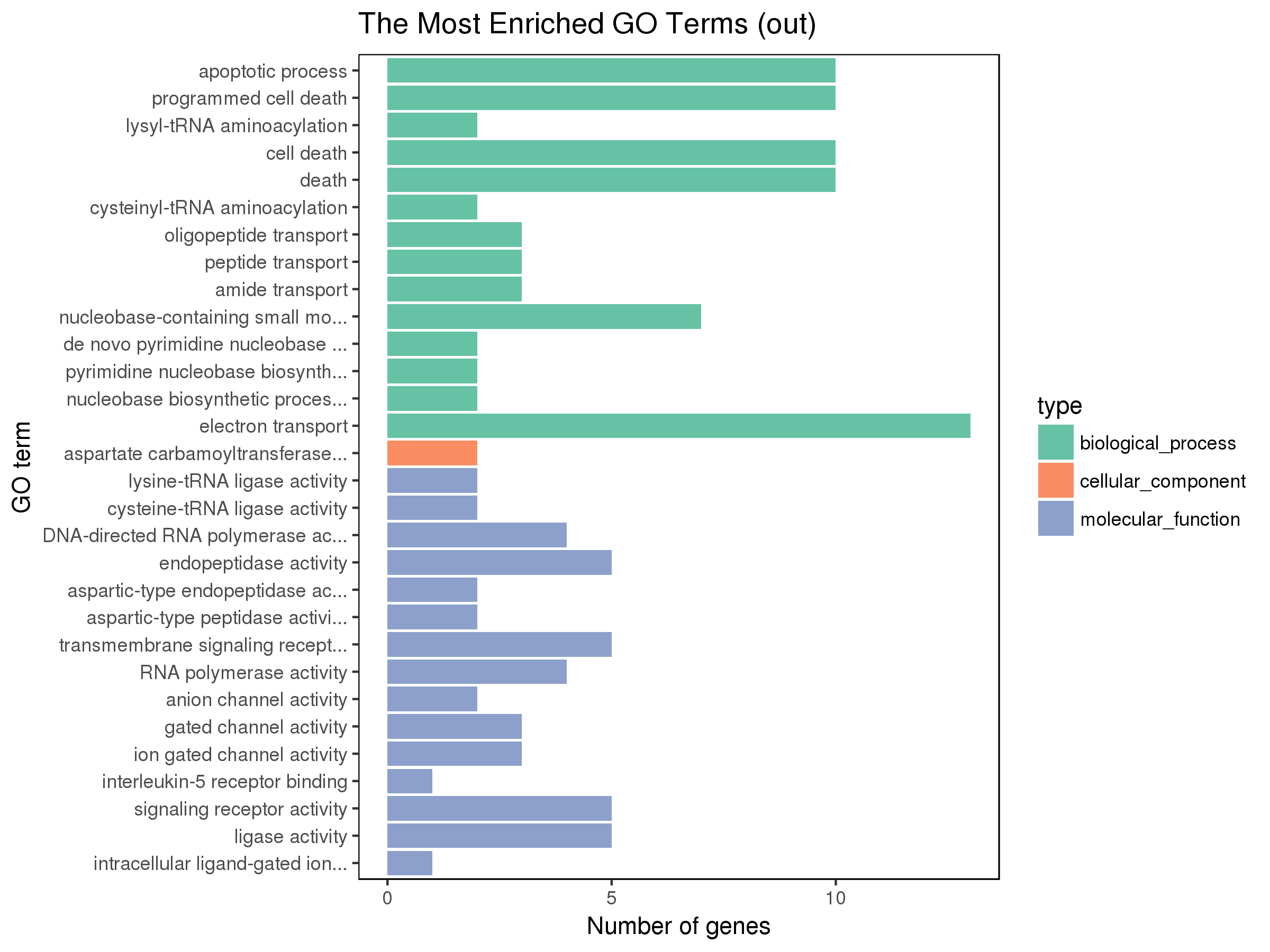

Supplement: Supplementary file 6 — Figure S6 [file EVA-13-2582-s006.png]

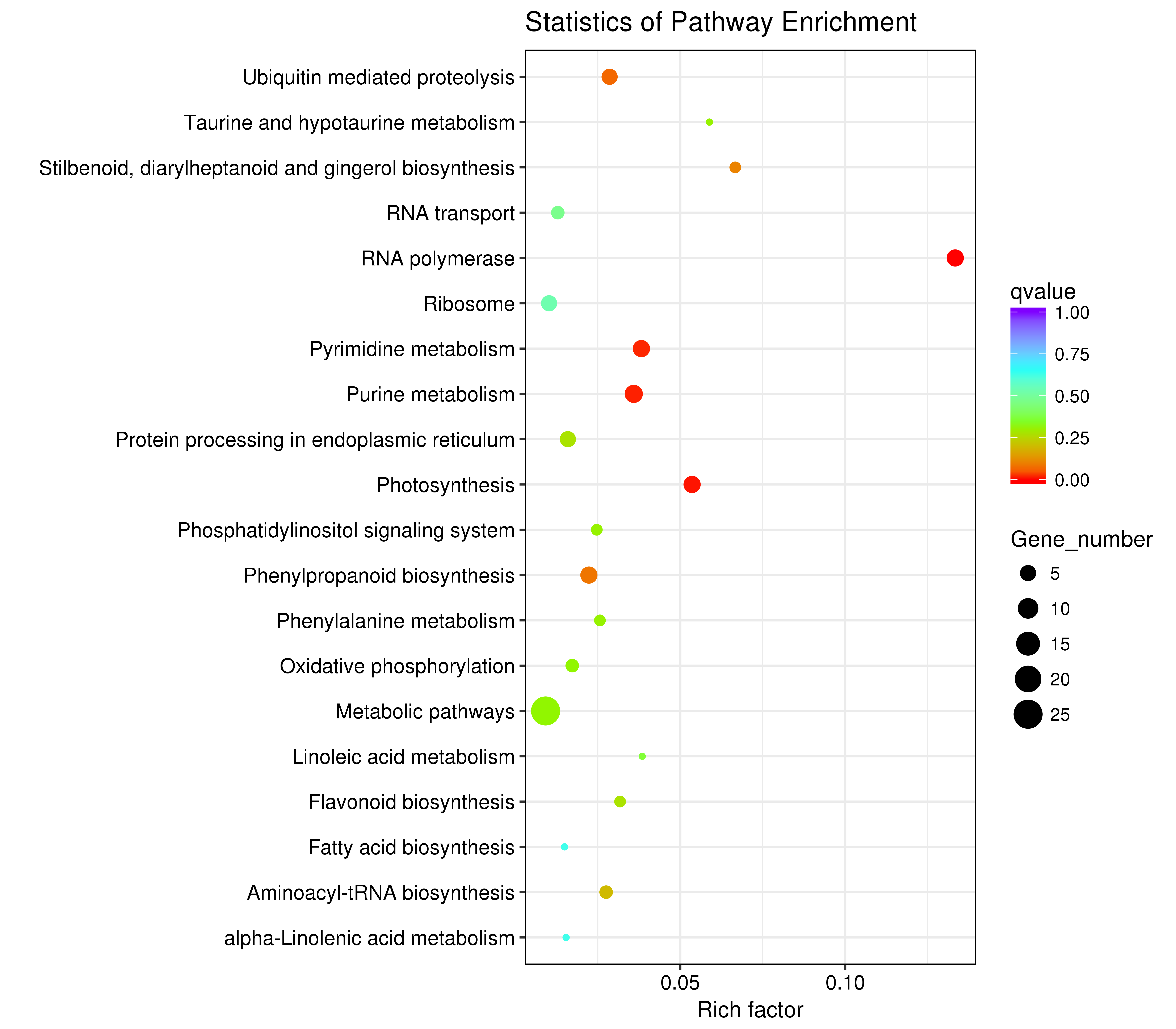

Supplement: Supplementary file 7 — Figure S7 [file EVA-13-2582-s007.png]

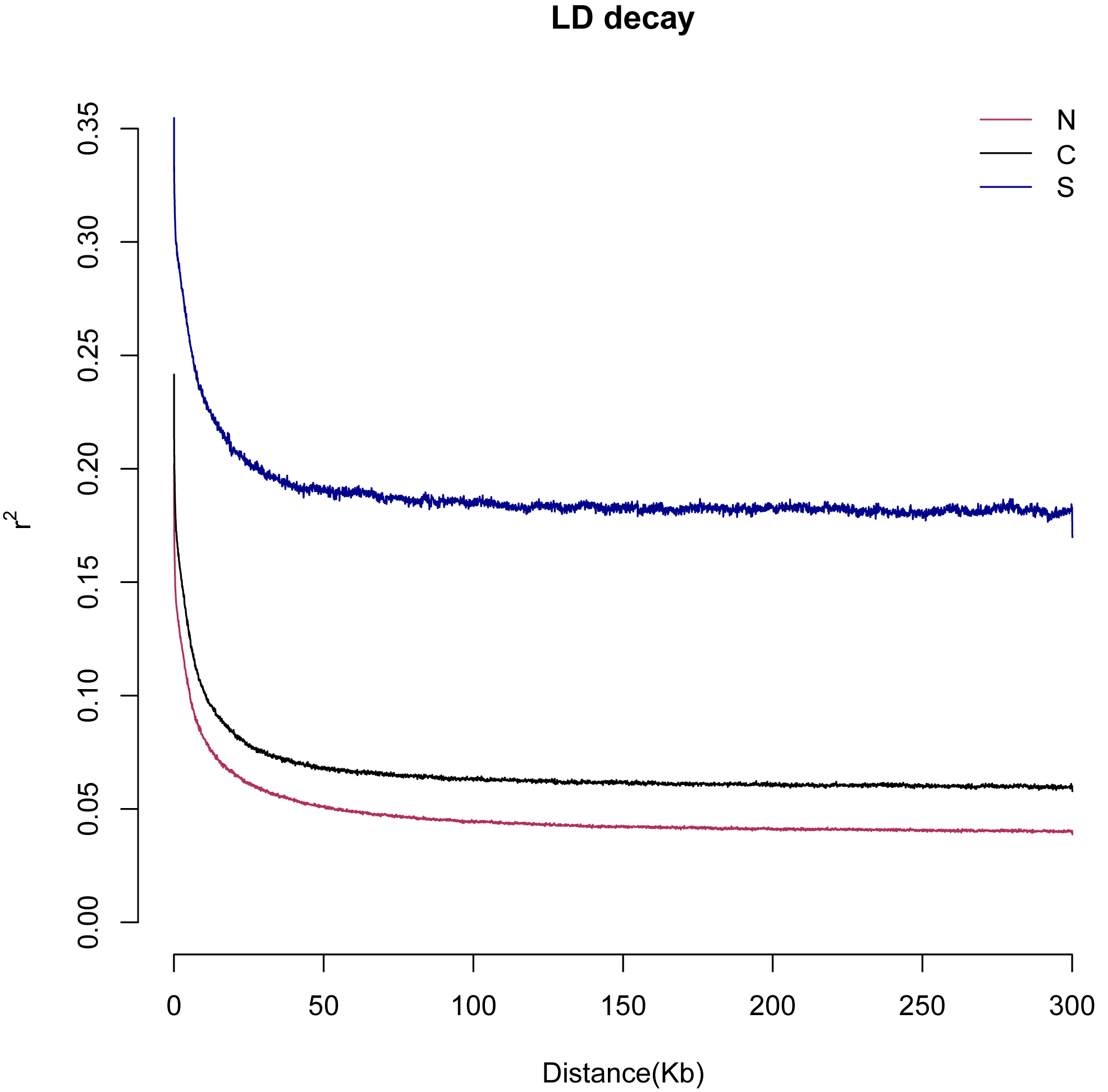

Supplement: Supplementary file 8 — Figure S8 [file EVA-13-2582-s008.tif]
