# Supplementary material for: Genetic architecture, demographic history, and genomic differentiation of Populus davidiana revealed by whole‐genome resequencing
Source: Evol Appl. 2020 Jul 15;13(10):2582–96. doi: 10.1111/eva.13046 (PMC7691461; doi:10.1111/eva.13046)
Supplement: Supplementary file 5 — Figure S5 [file EVA-13-2582-s005.pdf]

A1

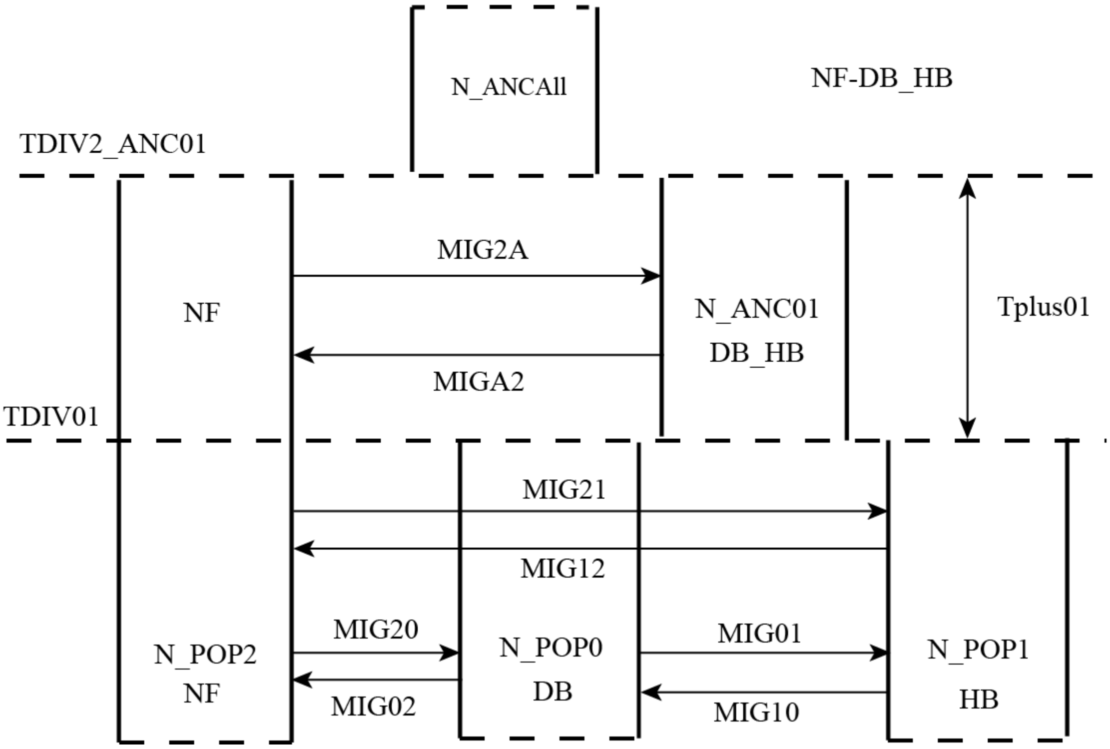
N_ANCAll
NF-DB_HB
TDIV2_ANC01
MIG2A
NF
N_ANC01
DB_HB
Tplus01
MIGA2
TDIV01
MIG21
MIG12
MIG20
N_POP2
NF
MIG02
N_POP0
DB
MIG01
MIG10
N_POP1
HB

A2

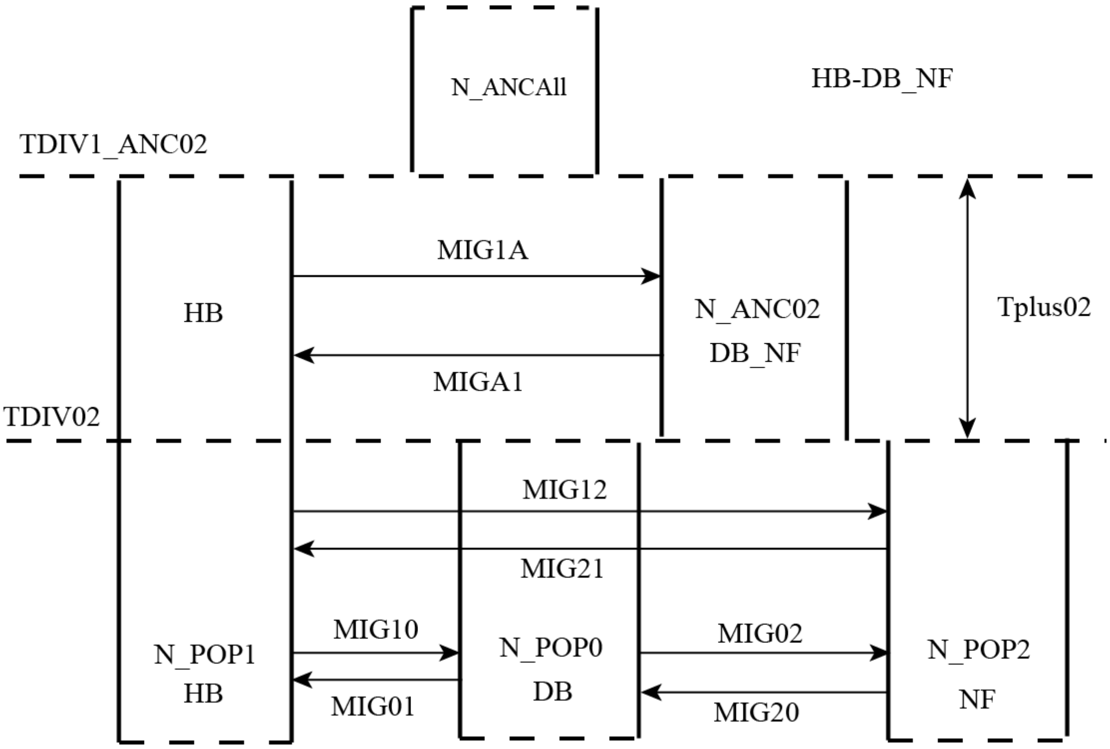
N_ANCAll
HB-DB_NF
TDIV1_ANC02
MIG1A
HB
N_ANC02
DB_NF
Tplus02
MIGA1
TDIV02
MIG12
MIG21
MIG10
N_POP1
HB
MIG01
N_POP0
DB
MIG02
MIG20
N_POP2
NF

A3

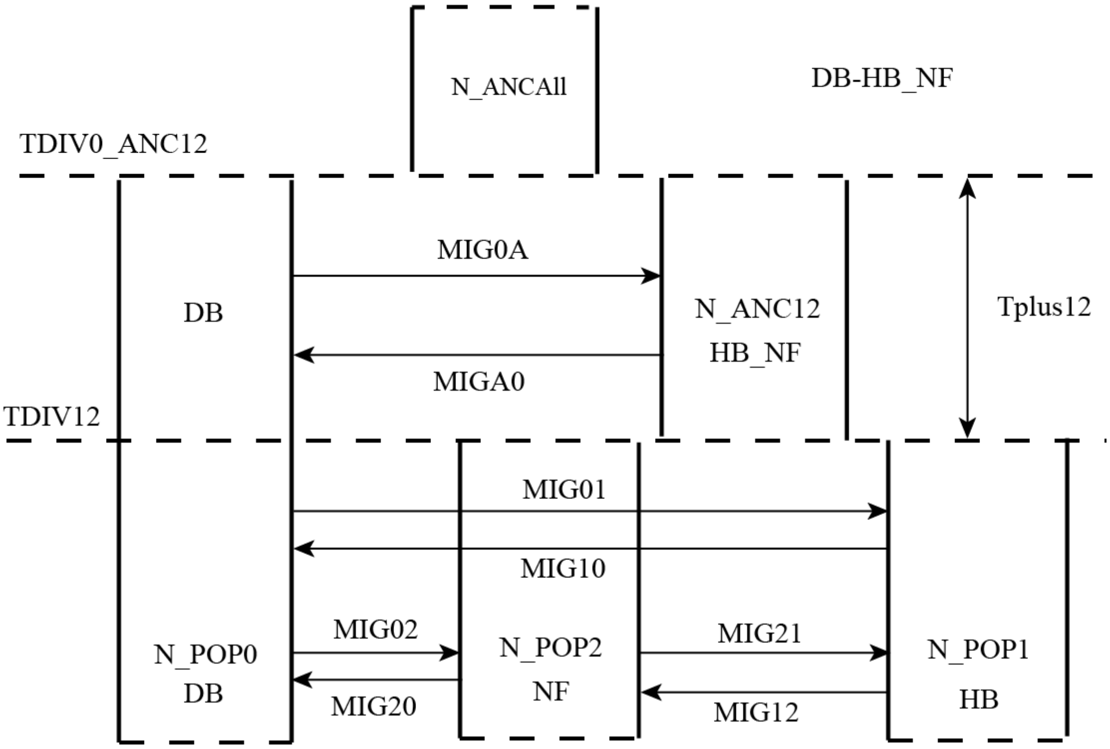

N_ANCAll
DB-HB_NF
TDIV0_ANC12
DB
MIG0A
MIGA0
N_ANC12
HB_NF
Tplus12
TDIV12
MIG01
MIG10
MIG02
MIG20
N_POP0
DB
N_POP2
NF
MIG21
MIG12
N_POP1
HB

A4

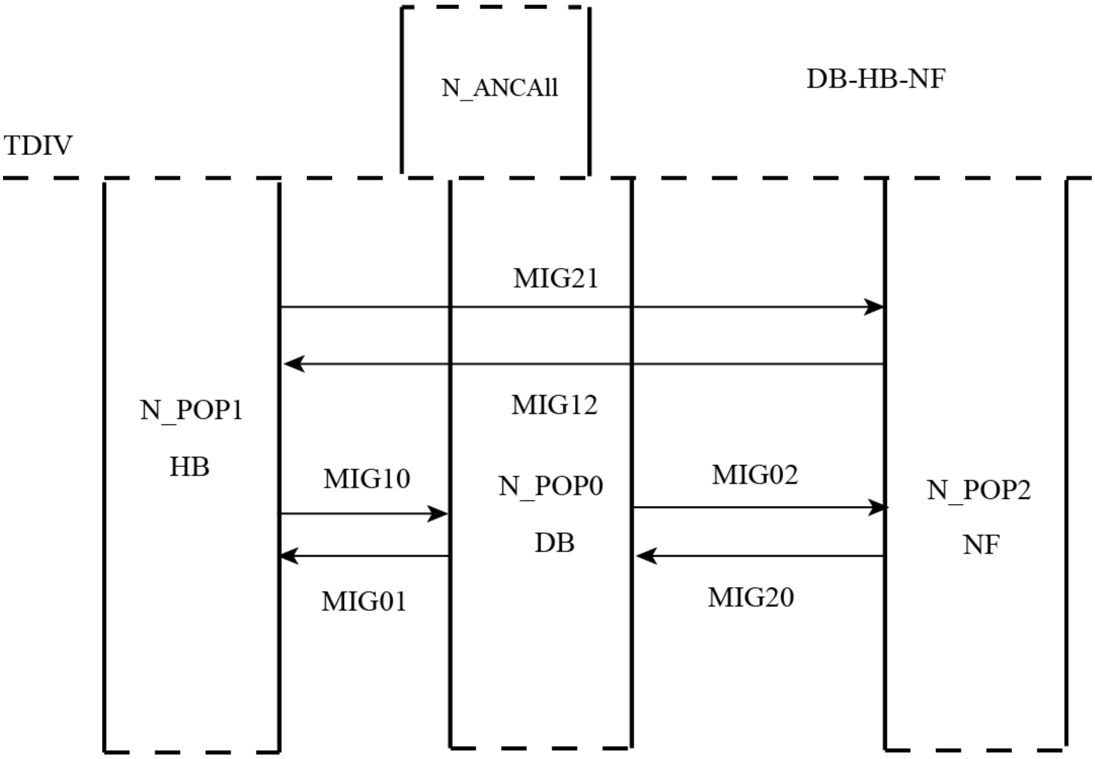

N_ANCAll
DB-HB-NF
TDIV
MIG21
MIG12
N_POP1
HB
MIG10
MIG01
N_POP0
DB
MIG02
MIG20
N_POP2
NF

B1

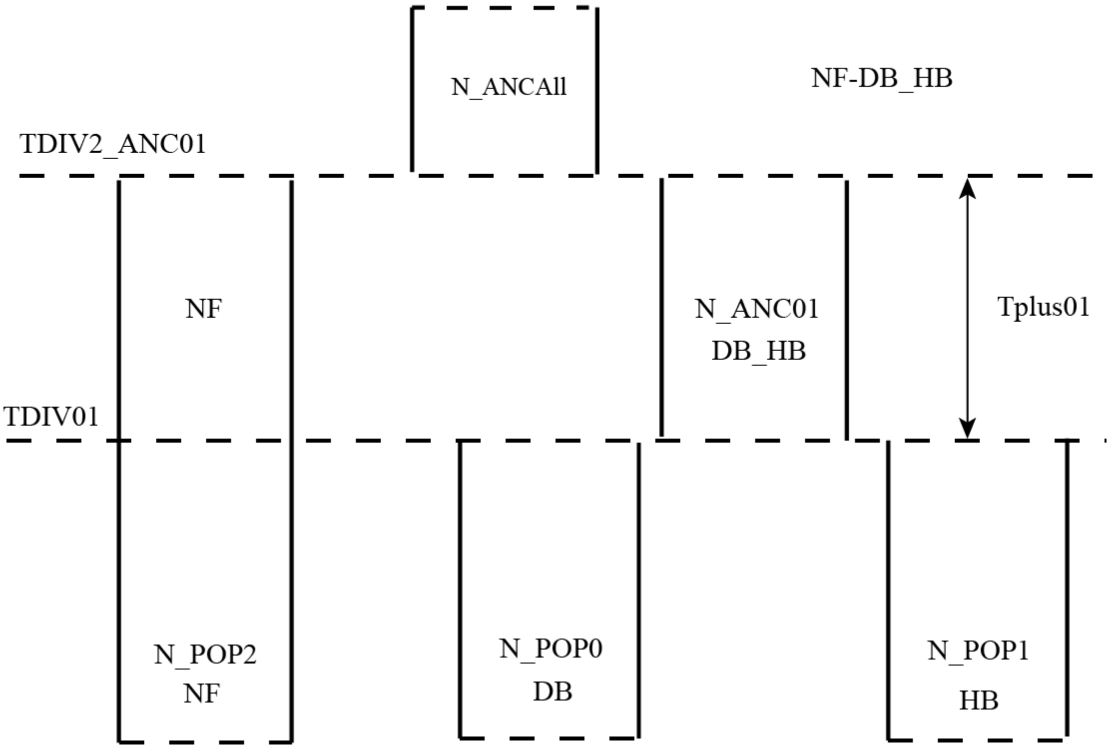
N_ANCAll
NF-DB_HB
TDIV2_ANC01
NF
N_ANC01
DB_HB
Tplus01
TDIV01
N_POP2
NF
N_POP0
DB
N_POP1
HB

B2

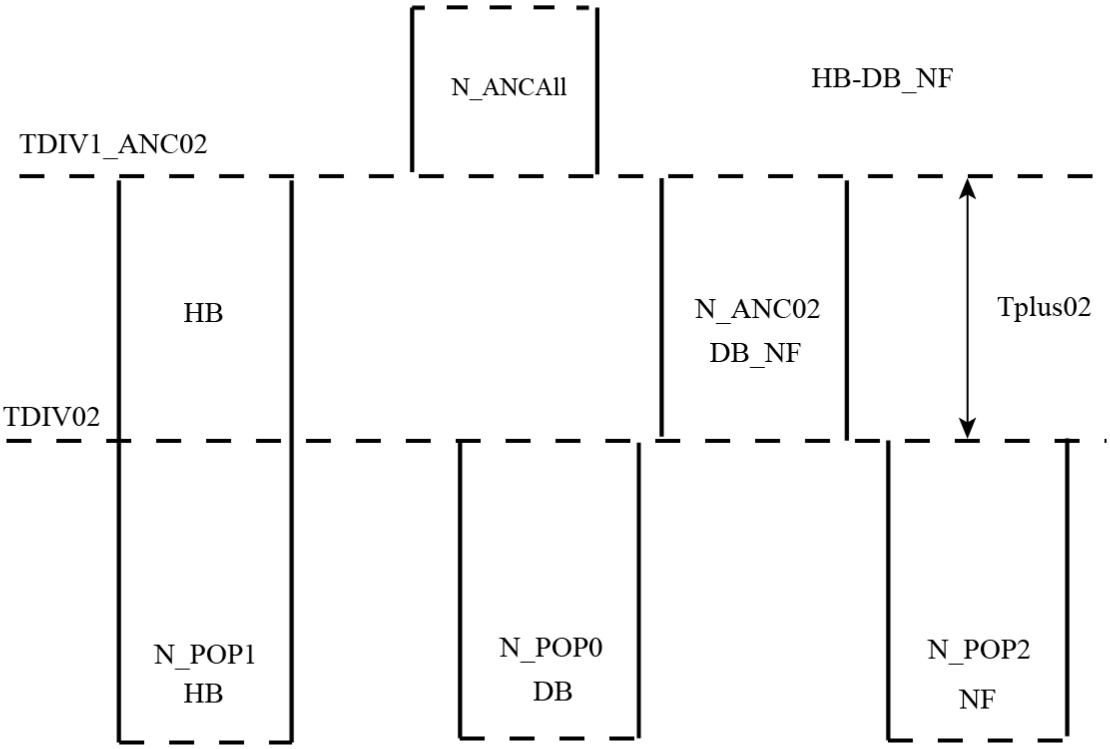
N_ANCAll
HB-DB_NF
TDIV1_ANC02
HB
N_ANC02
DB_NF
Tplus02
TDIV02
N_POP1
HB
N_POP0
DB
N_POP2
NF

B3

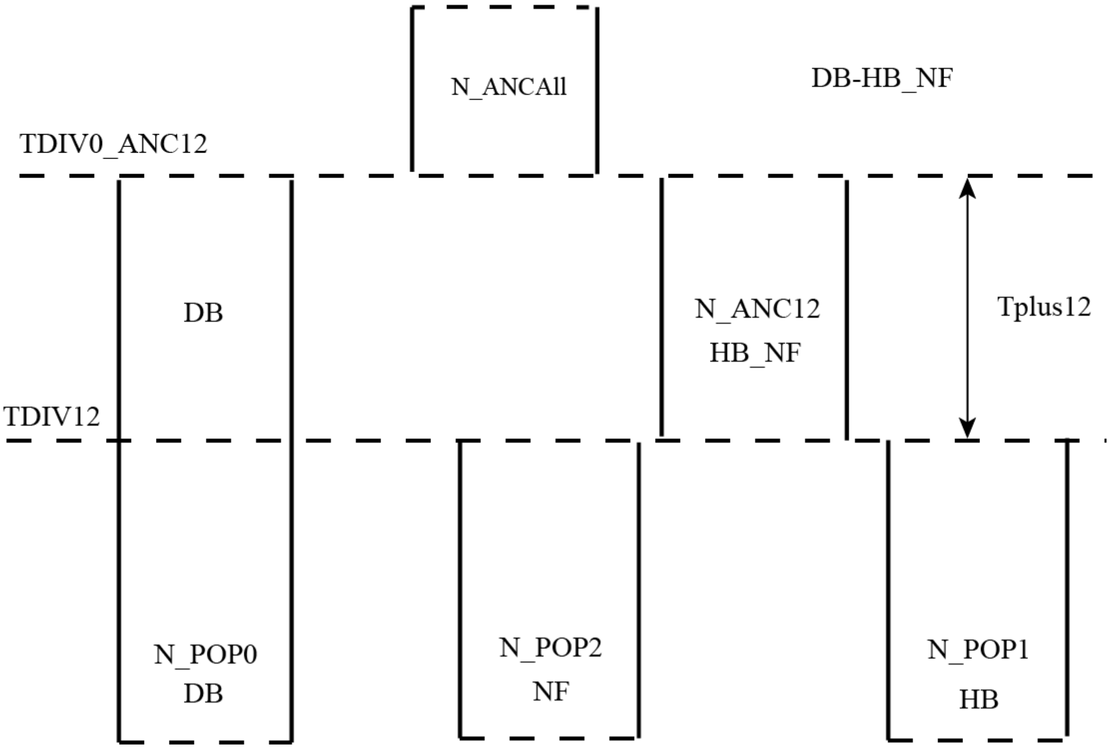
N_ANCAll
DB-HB_NF
TDIV0_ANC12
DB
N_ANC12
HB_NF
Tplus12
TDIV12
N_POP0
DB
N_POP2
NF
N_POP1
HB

B4

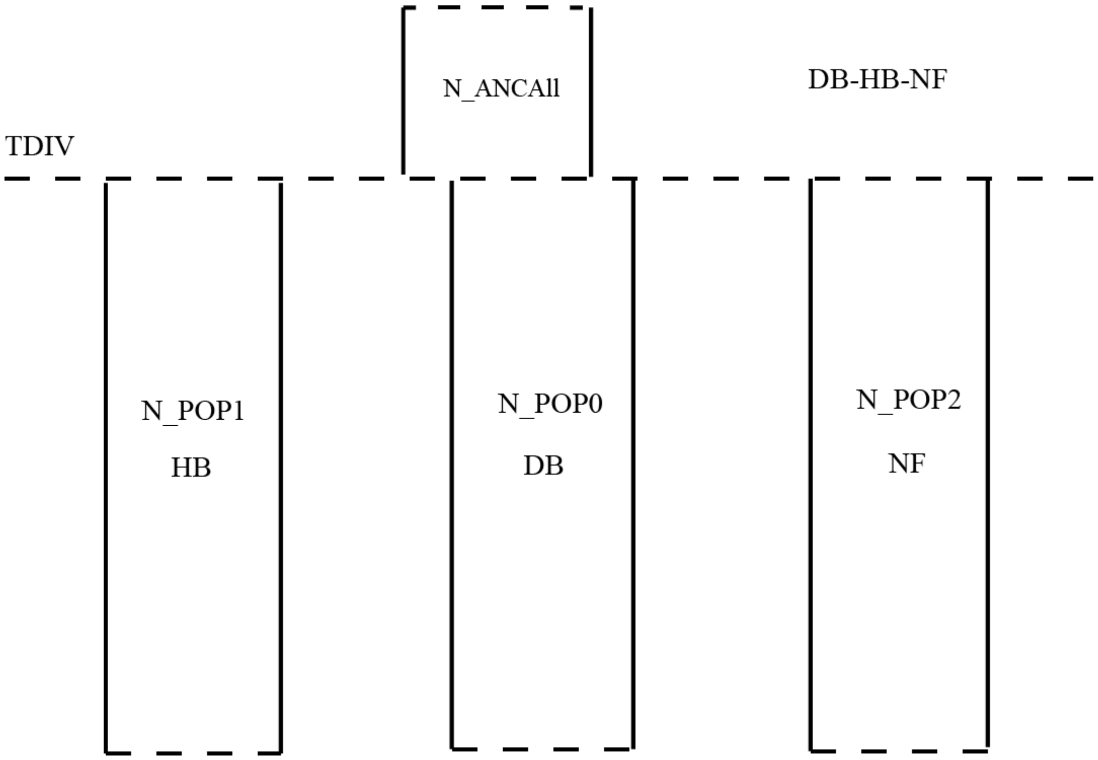
N_ANCAll
DB-HB-NF
TDIV
N_POP1
HB
N_POP0
DB
N_POP2
NF

C1

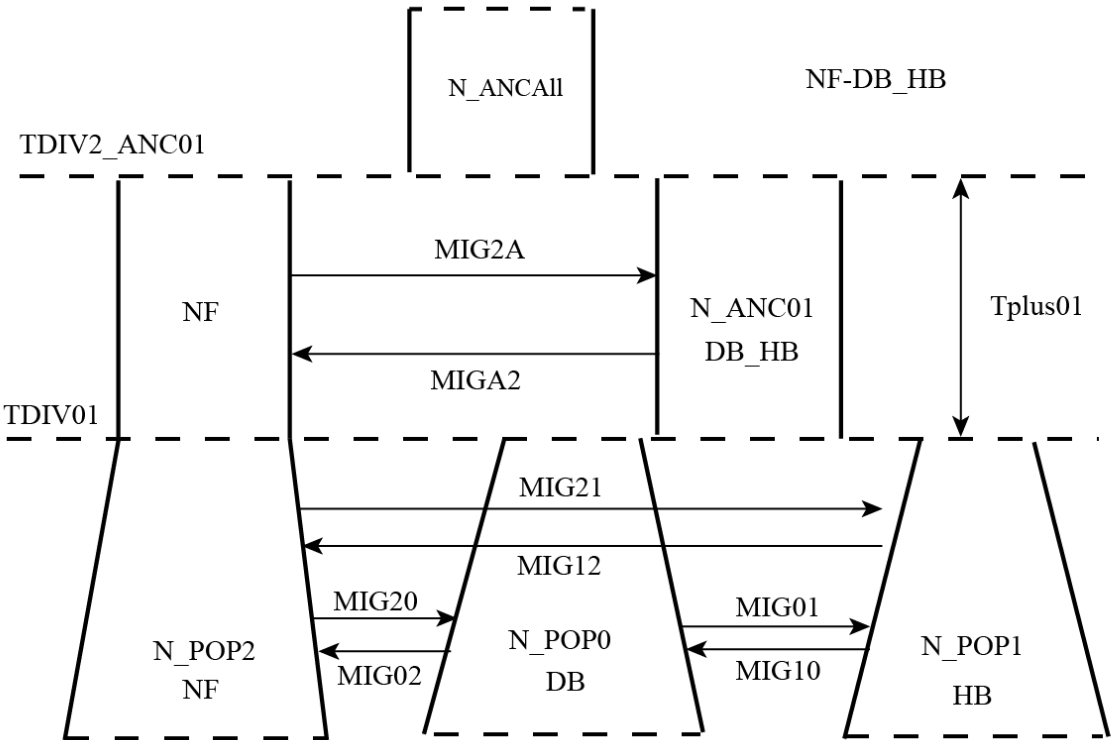
N_ANCAll
NF-DB_HB
TDIV2_ANC01
NF
MIG2A
MIGA2
N_ANC01
DB_HB
Tplus01
TDIV01
MIG21
MIG12
MIG20
MIG02
MIG01
MIG10
N_POP2
NF
N_POP0
DB
N_POP1
HB

C2

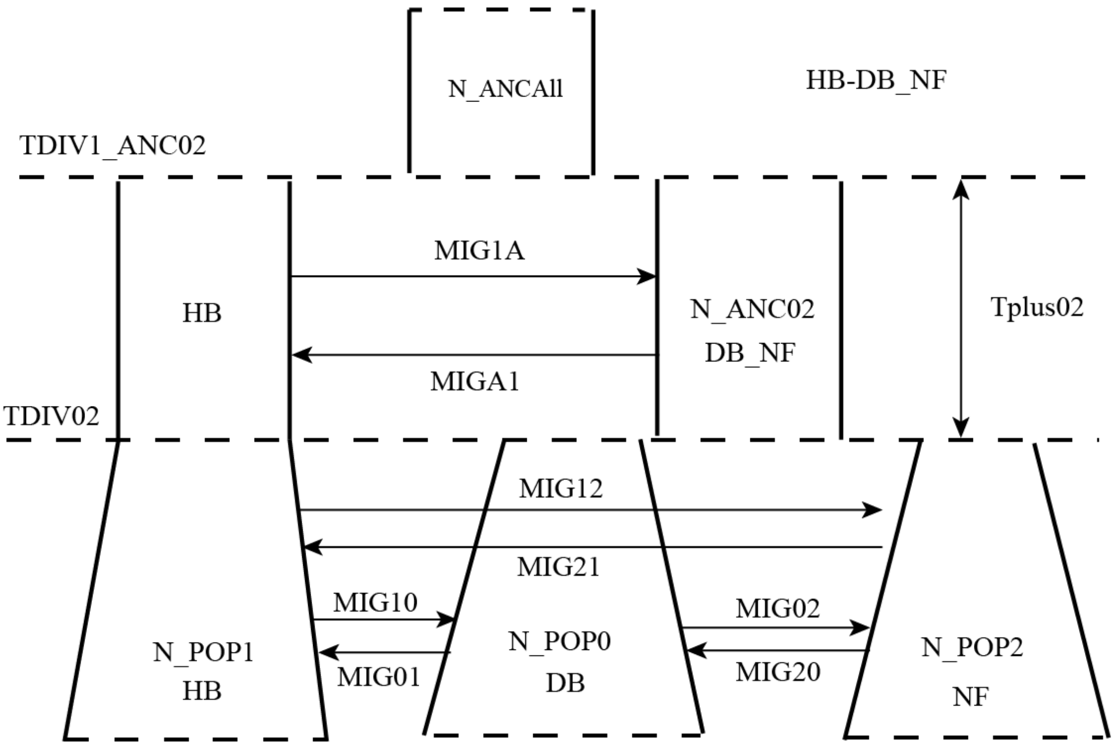
N_ANCAll
HB-DB_NF
TDIV1_ANC02
HB
MIG1A
MIGA1
N_ANC02
DB_NF
Tplus02
TDIV02
MIG12
MIG21
MIG10
MIG01
MIG02
MIG20
N_POP1
HB
N_POP0
DB
N_POP2
NF

C3

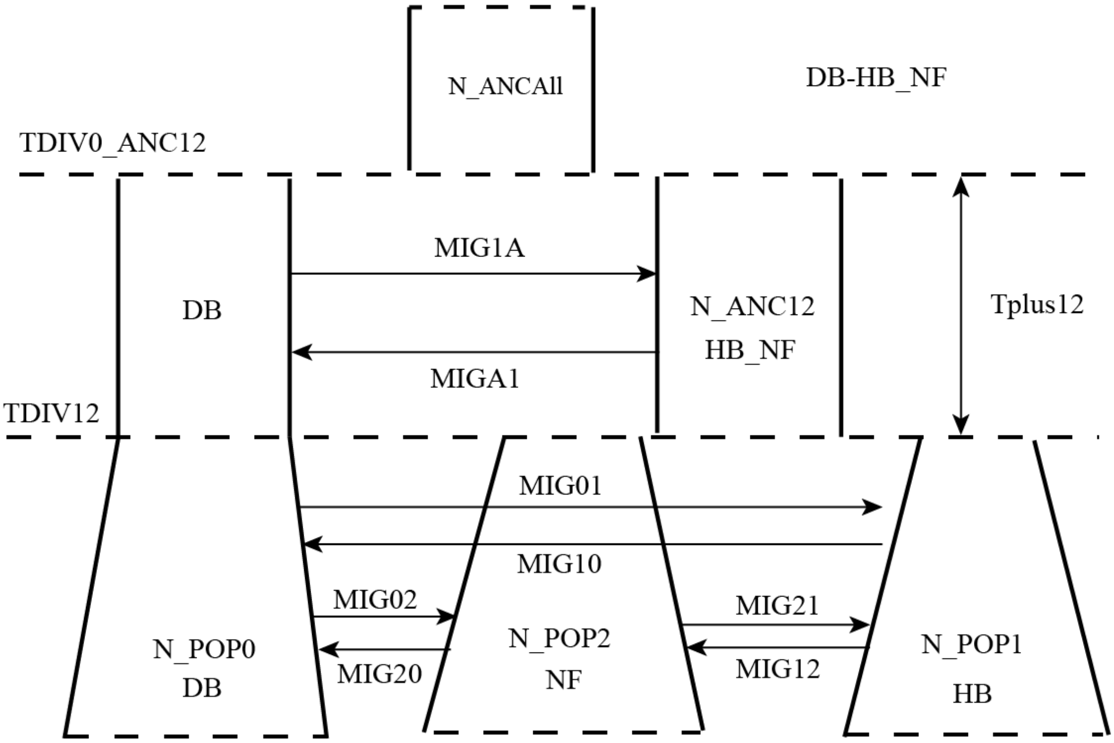

N_ANCAll
DB-HB_NF
TDIV0_ANC12
MIG1A
DB
N_ANC12
HB_NF
Tplus12
MIGA1
TDIV12
MIG01
MIG10
MIG02
MIG21
N_POP2
NF
N_POP0
DB
MIG20
MIG12
N_POP1
HB

C4

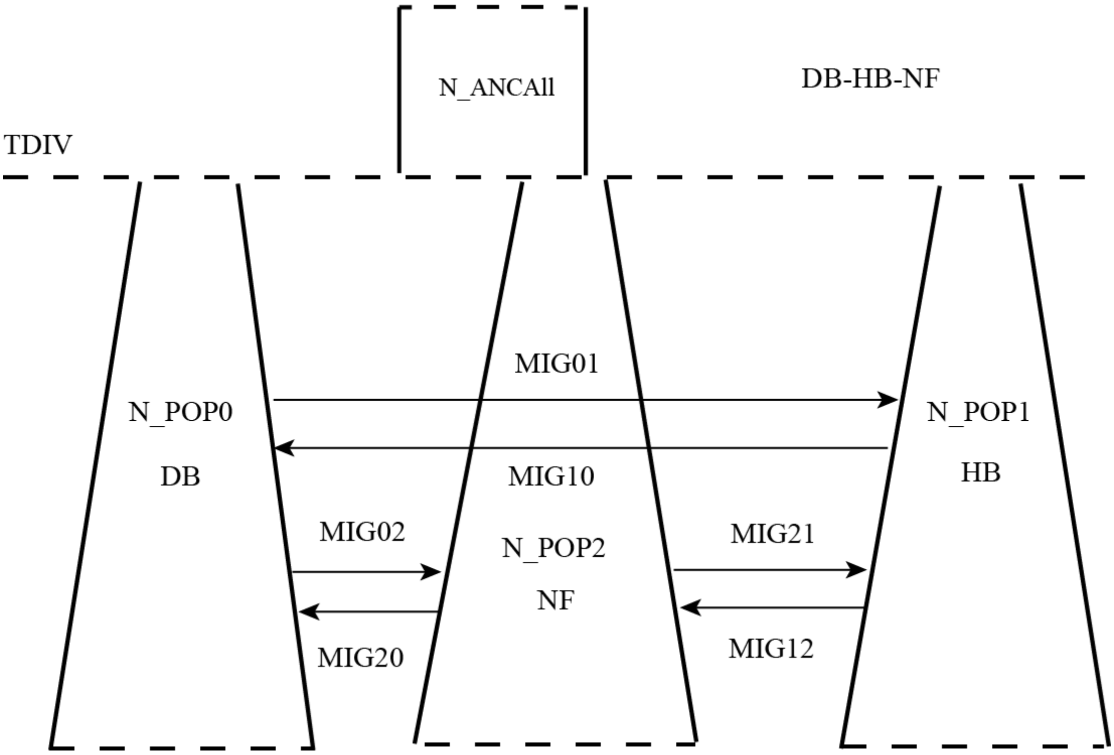

N_ANCAll
DB-HB-NF
TDIV
MIG01
N_POP0
DB
N_POP1
HB
MIG10
MIG02
N_POP2
NF
MIG21
MIG20
MIG12

D1

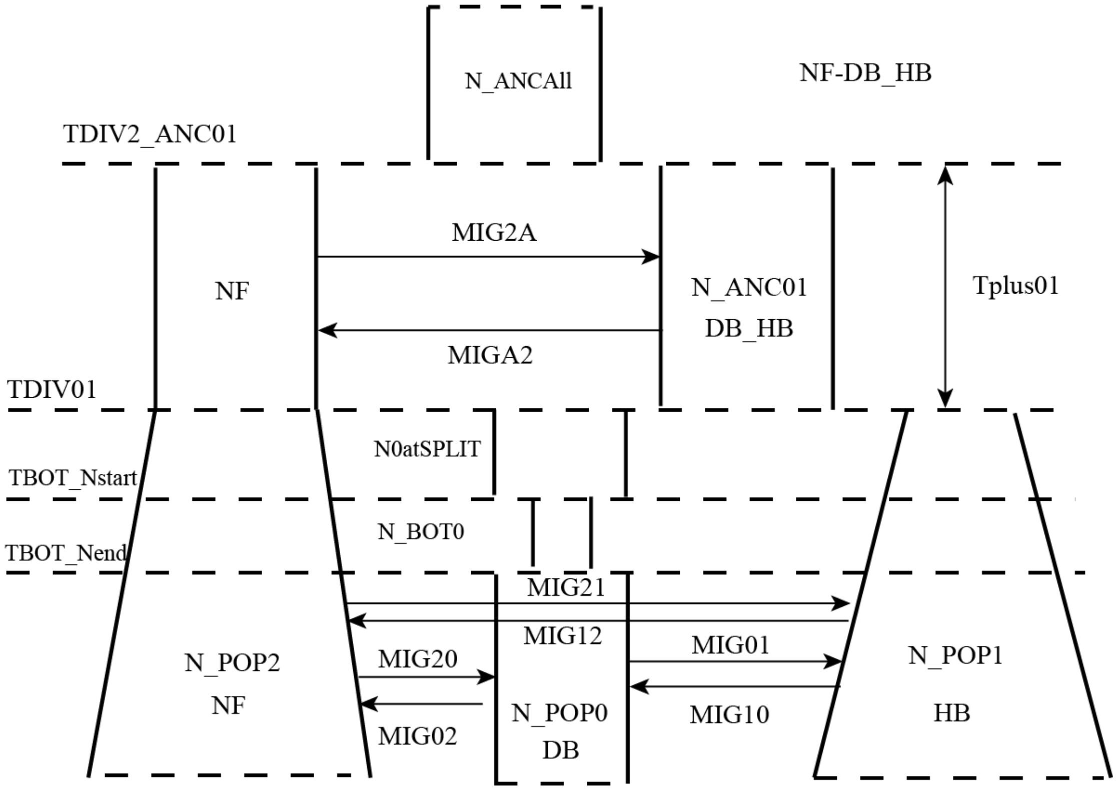
N_ANCAll
NF-DB_HB
TDIV2_ANC01
MIG2A
NF
N_ANC01
DB_HB
Tplus01
MIGA2
TDIV01
N0atSPLIT
TBOT_Nstart
N_BOT0
TBOT_Nend
MIG21
MIG12
MIG20
MIG01
N_POP2
NF
N_POP1
HB
N_POP0
DB
MIG02
MIG10

D2

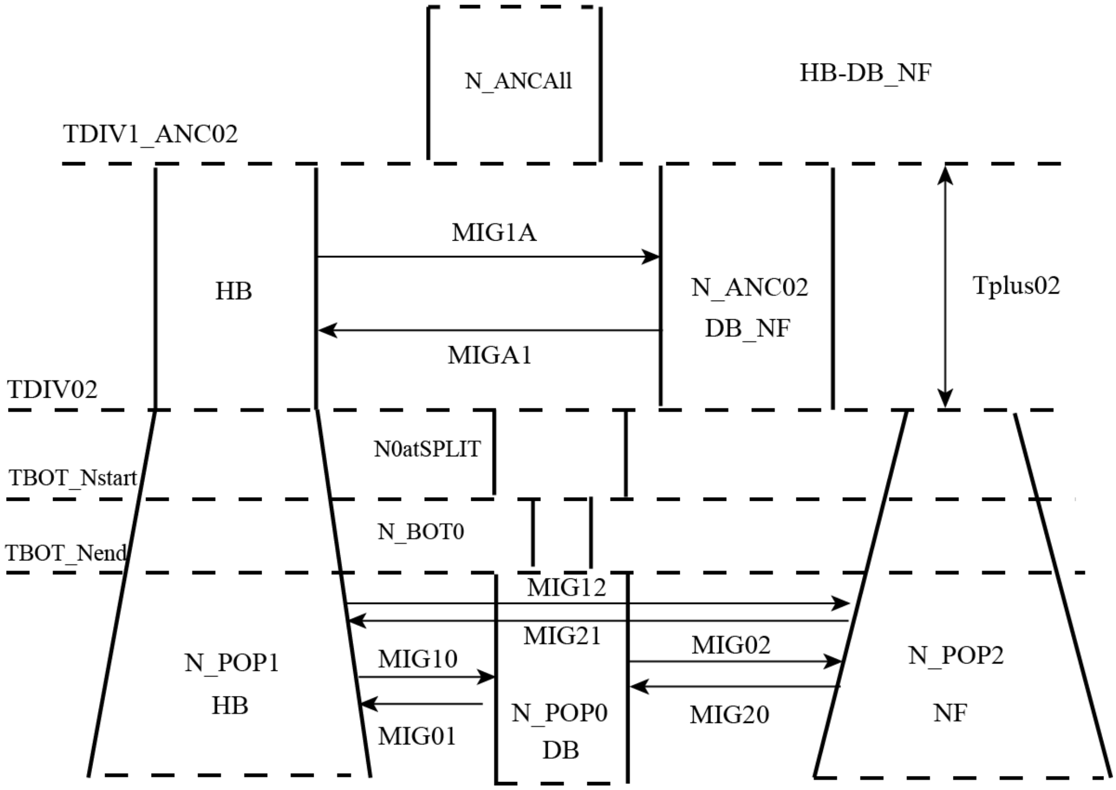
N_ANCAll
HB-DB_NF
TDIV1_ANC02
MIG1A
HB
N_ANC02
DB_NF
Tplus02
MIGA1
TDIV02
N0atSPLIT
TBOT_Nstart
N_BOT0
TBOT_Nend
MIG12
MIG21
MIG10
MIG02
N_POP1
HB
N_POP2
NF
N_POP0
DB
MIG01
MIG20

D3

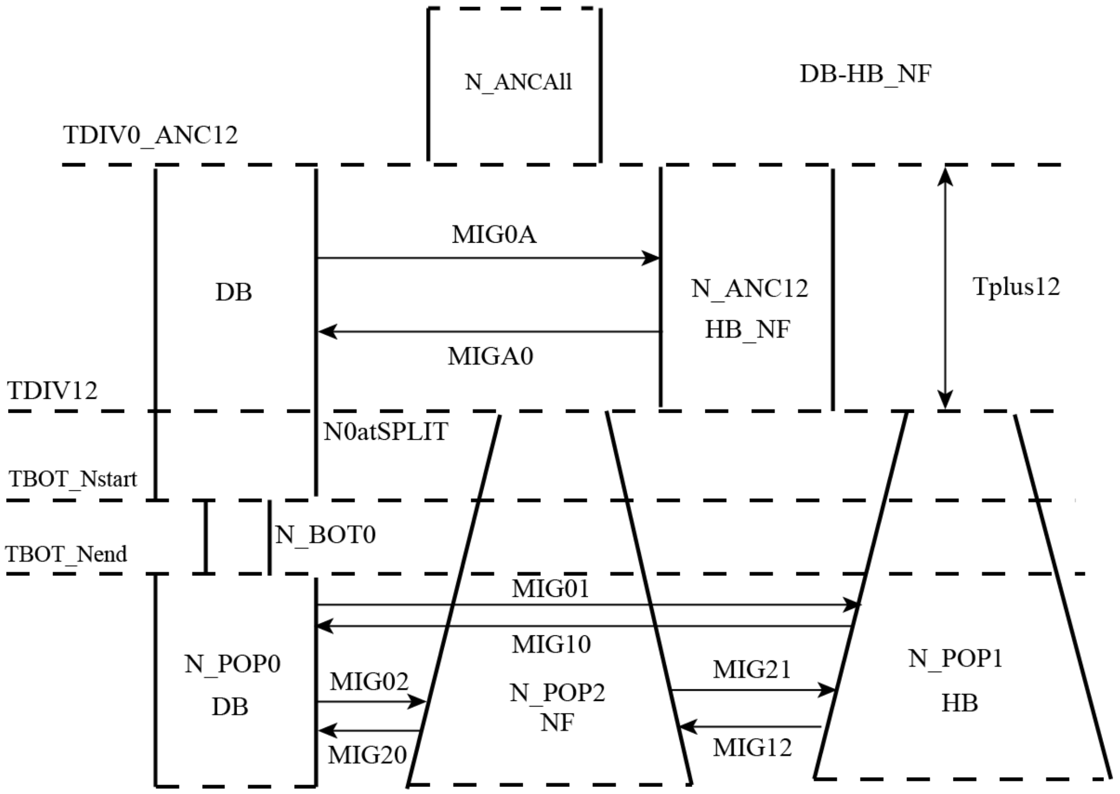
N_ANCAll
DB-HB_NF
TDIV0_ANC12
MIG0A
DB
N_ANC12
HB_NF
Tplus12
MIGA0
TDIV12
N0atSPLIT
TBOT_Nstart
N_BOT0
TBOT_Nend
MIG01
MIG10
N_POP0
DB
MIG02
N_POP2
NF
MIG21
N_POP1
HB
MIG20
MIG12

D4

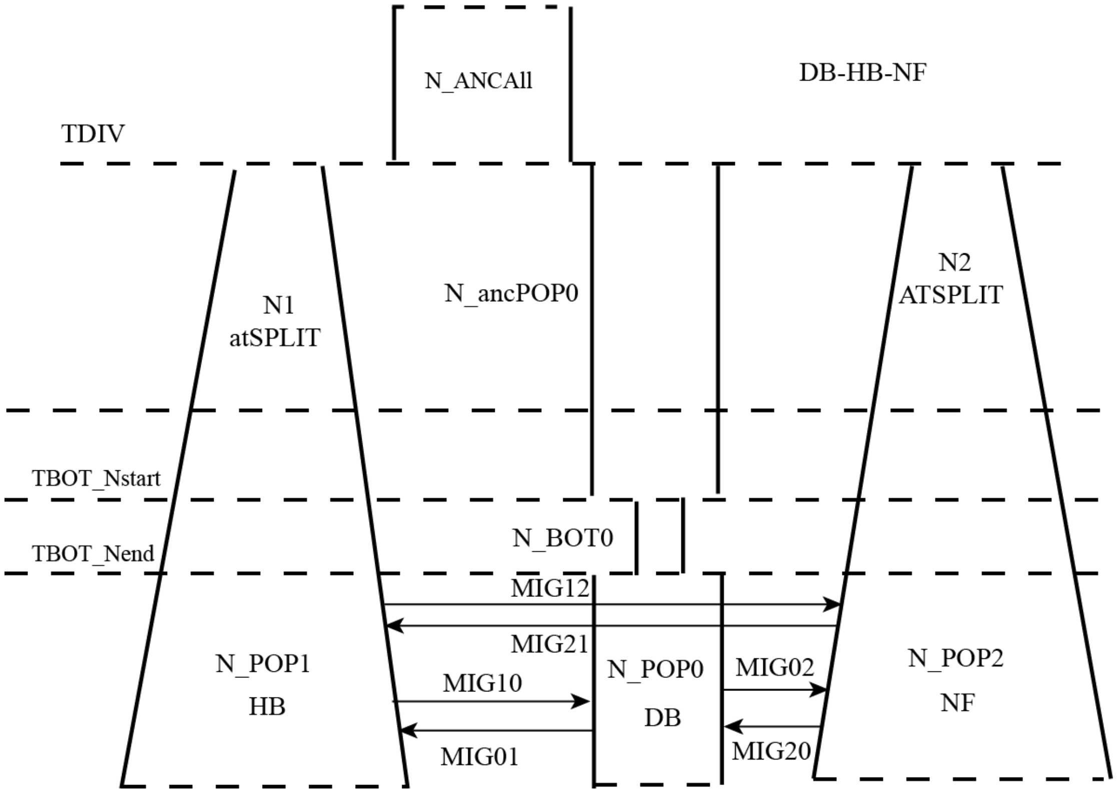
N_ANCAll
DB-HB-NF
TDIV
N1
atSPLIT
N_ancPOP0
N2
ATSPLIT
TBOT_Nstart
N_BOT0
TBOT_Nend
MIG12
MIG21
N_POP1
HB
MIG10
N_POP0
DB
MIG02
N_POP2
NF
MIG01
MIG20

F1

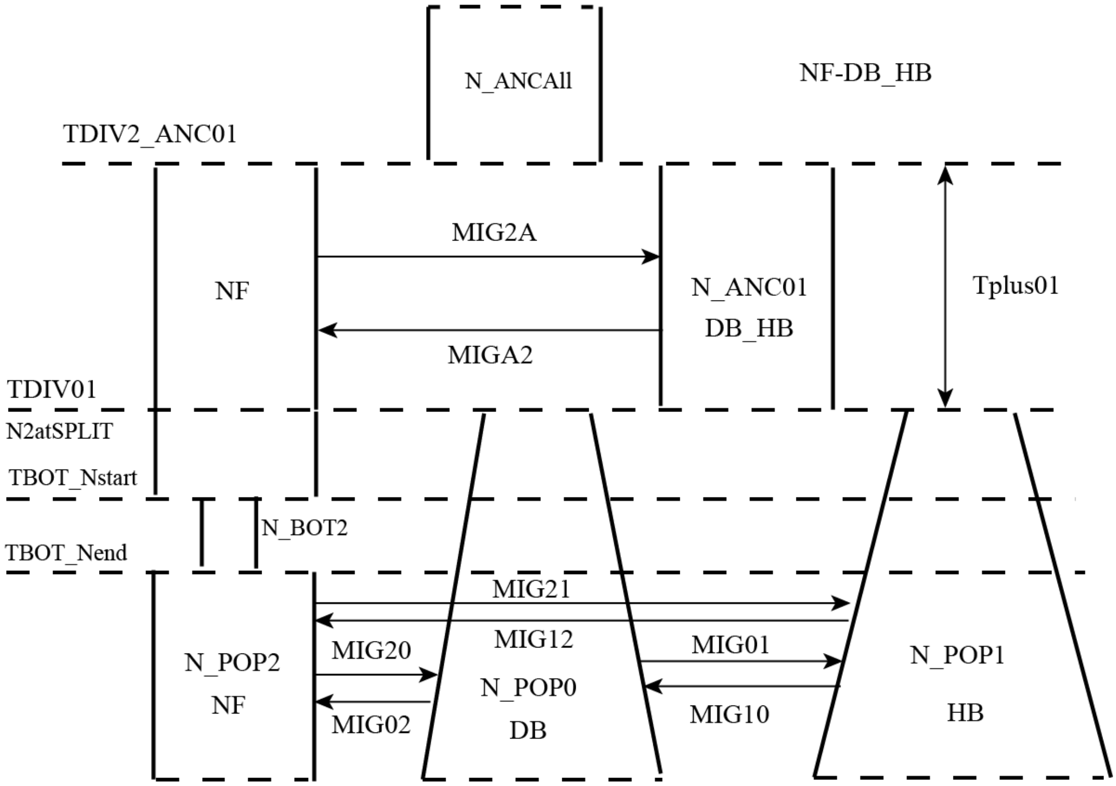
N_ANCAll
NF-DB_HB
TDIV2_ANC01
MIG2A
NF
N_ANC01
DB_HB
Tplus01
MIGA2
TDIV01
N2atSPLIT
TBOT_Nstart
N_BOT2
TBOT_Nend
MIG21
MIG12
MIG20
N_POP2
NF
MIG01
N_POP1
HB
N_POP0
DB
MIG02
MIG10

F2

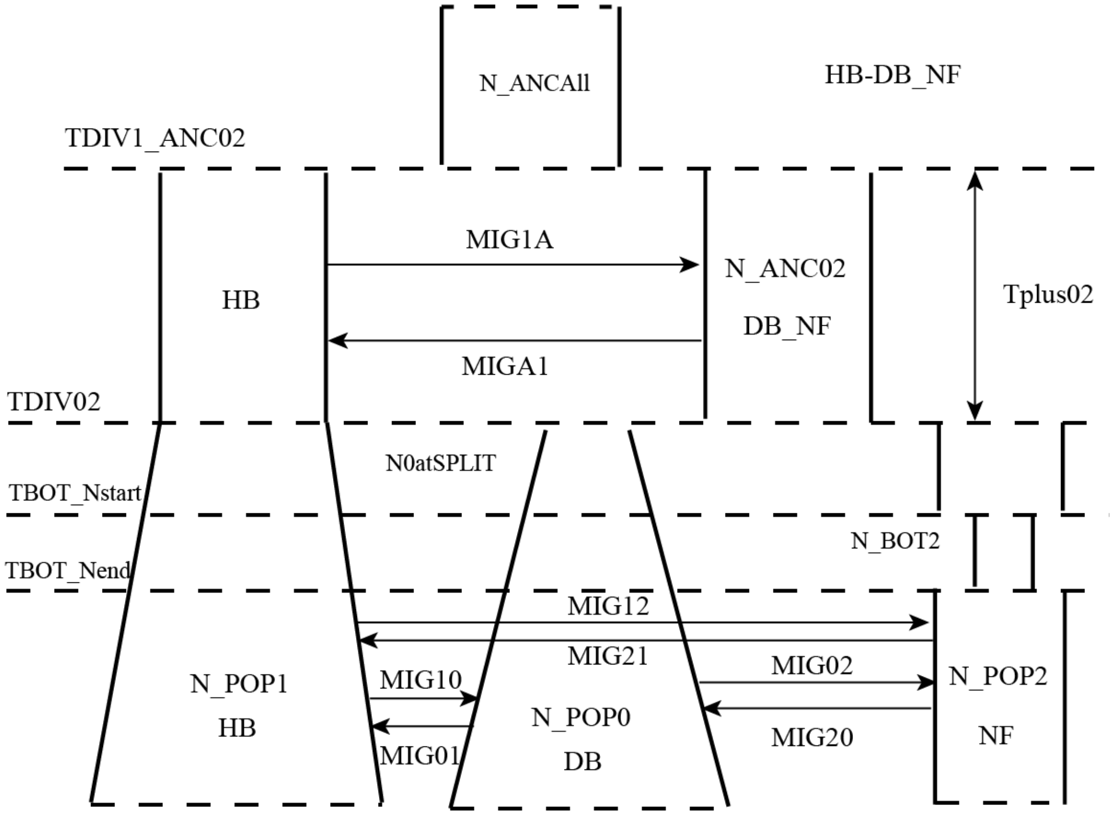
N_ANCAll
HB-DB_NF
TDIV1_ANC02
MIG1A
HB
N_ANC02
DB_NF
Tplus02
MIGA1
TDIV02
N0atSPLIT
TBOT_Nstart
N_BOT2
TBOT_Nend
MIG12
MIG21
MIG02
N_POP1
HB
MIG10
N_POP2
NF
N_POP0
DB
MIG01
MIG20

F3

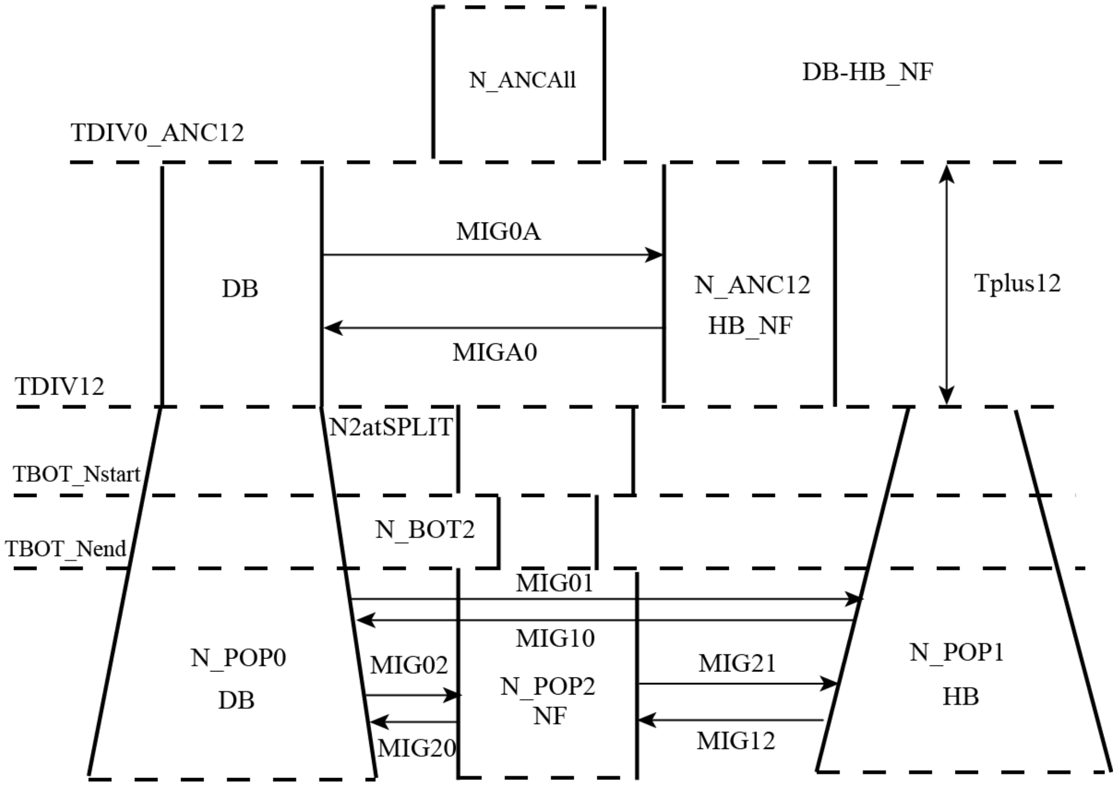

F4

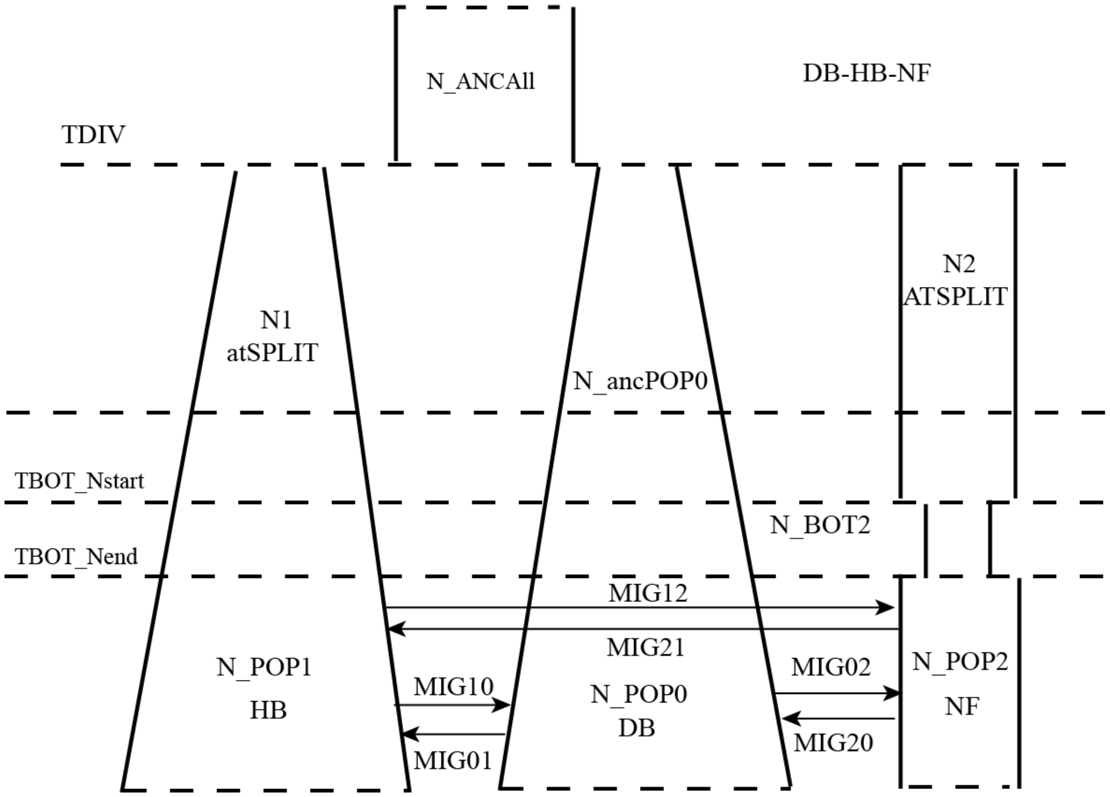

Figure S5. 20 divergence scenarios tested in FASTSIMCOAL2. A : AsymmetricMigration without population expansion. (A1) A bifurcation model in which DB and HB are sister taxa. (A2) A bifurcation model in which DB and NF are

sister taxa. (A3) A bifurcation model in which HB and NF are sister taxa. (A4) A trifurcation model in which all three populations diverged simultaneously from a common ancestor. B: NoMigration. (B1) A bifurcation model in which DB and HB are sister taxa. (B2) A bifurcation model in which DB and NF are sister taxa. (B3) A bifurcation model in which HB and NF are sister taxa. (B4) A trifurcation model in which all three populations diverged simultaneously from a common ancestor. C: complex model, which includes a bottleneck in DB. (C1) A bifurcation model in which DB and HB are sister taxa. (C2) A bifurcation model in which DB and NF are sister taxa. (C3) A bifurcation model in which HB and NF are sister taxa. (C4) A trifurcation model in which all three populations diverged simultaneously from a common ancestor. D: complex model, which includes a bottleneck in NF. (D1) A bifurcation model in which DB and HB are sister taxa. (D2) A bifurcation model in which DB and NF are sister taxa. (D3) A bifurcation model in which HB and NF are sister taxa. (D4) A trifurcation model in which all three populations diverged simultaneously from a common ancestor.
