# Supplementary material for: Genetic architecture, demographic history, and genomic differentiation of Populus davidiana revealed by whole‐genome resequencing
Source: Evol Appl. 2020 Jul 15;13(10):2582–96. doi: 10.1111/eva.13046 (PMC7691461; doi:10.1111/eva.13046)
Supplement: Supplementary file 9 — Tables S1‐S4 [file EVA-13-2582-s009.docx]

**Appendix**

Additional information may be found in the online version of this article.

Table S1 Summary statistics of Illumina re-sequencing data per sample

| Sample ID | Location | Altitude（m） | Longitude | Latitude | Mapping rate | Mean Coverage |
| --- | --- | --- | --- | --- | --- | --- |
| SYS30 | Shuangyashan | 179 | E 131°15′ | N 46°64′ | 92.36% | 30.50 |
| SYS33 | Shuangyashan | 179 | E 131°15′ | N 46°64′ | 90.25% | 33.70 |
| HN2 | Huanan | 177 | E 130°55′ | N 46°23′ | 90.08% | 40.58 |
| HN9 | Huanan | 177 | E 130°55′ | N 46°23′ | 90.78% | 32.25 |
| HH1 | Heihe | 122 | E 127°52′ | N 50°24′ | 91.25% | 34.23 |
| HH2 | Heihe | 122 | E 127°52′ | N 50°24′ | 92.36% | 30.08 |
| HH12 | Heihe | 122 | E 127°52′ | N 50°24′ | 88.08% | 31.25 |
| HH16 | Heihe | 122 | E 127°52′ | N 50°24′ | 89.98% | 30.25 |
| HH23 | Heihe | 122 | E 127°52′ | N 50°24′ | 90.05% | 35.62 |
| LHS3 | Lianhuashan | 215 | E 124°49′ | N 44°47′ | 93.25% | 36.28 |
| LHS8 | Lianhuashan | 215 | E 124°49′ | N 44°47′ | 89.28% | 33.36 |
| LHS14 | Lianhuashan | 215 | E 124°49′ | N 44°47′ | 88.89% | 32.29 |
| LHS15 | Lianhuashan | 215 | E 124°49′ | N 44°47′ | 90.21% | 36.68 |
| SS1 | Shuangshan | 130 | E 123°88′ | N 43°68′ | 93.25% | 30.05 |
| SS15 | Shuangshan | 130 | E 123°88′ | N 43°68′ | 88.08% | 32.03 |
| SS18 | Shuangshan | 130 | E 123°88′ | N 43°68′ | 90.78% | 31.05 |
| SS24 | Shuangshan | 130 | E 123°88′ | N 43°68′ | 92.58% | 30.89 |
| HM8 | Huma | 170 | E 126°66′ | N 51°72′ | 90.32% | 35.62 |
| HM18 | Huma | 170 | E 126°66′ | N 51°72′ | 91.25% | 32.05 |
| HM21 | Huma | 170 | E 126°66′ | N 51°72′ | 93.25% | 30.06 |
| BSS1 | Baishishan | 346 | E 127°55′ | N 43°58′ | 90.04% | 32.89 |
| BSS2 | Baishishan | 346 | E 127°55′ | N 43°58′ | 88.28% | 30.04 |
| BSS15 | Baishishan | 346 | E 127°55′ | N 43°58′ | 90.87% | 35.21 |
| BSS18 | Baishishan | 346 | E 127°55′ | N 43°58′ | 90.07% | 32.78 |
| BSS20 | Baishishan | 346 | E 127°55′ | N 43°58′ | 88.98% | 30.07 |
| JSZ17 | Jinshazhen | 270 | E 126°79′ | N 43°15′ | 88.88% | 31.05 |
| JSZ18 | Jinshazhen | 270 | E 126°79′ | N 43°15′ | 90.58% | 31.23 |
| JSZ22 | Jinshazhen | 270 | E 126°79′ | N 43°15′ | 90.41% | 30.08 |
| JSZ25 | Jinshazhen | 270 | E 126°79′ | N 43°15′ | 90.58% | 31.56 |
| WS2 | Wushan | 220 | E 109°87′ | N 31°07′ | 89.08% | 30.09 |
| WC2 | Weichang | 847 | E 117°76′ | N 41°93′ | 89.23% | 35.02 |
| WT2 | Wutai | 1032 | E 113°25′ | N 38°72′ | 88.78% | 40.25 |
| HL2 | Hualin | 52 | E 116°40′ | N 39°90′ | 88.96% | 38.02 |
| WT16 | Wutai | 1032 | E 113°25′ | N 38°72′ | 89.98% | 34.25 |
| HL1 | Hualin | 52 | E 116°40′ | N 39°90′ | 90.25% | 33.32 |
| BTM17 | Baotianman | 1182 | E 111°92′ | N 33°49′ | 90.24% | 31.05 |
| WC1 | Weichang | 847 | E 117°76′ | N 41°93′ | 89.98% | 32.05 |
|  |  |  |  |  |  |  |
| Sample ID | Location | Altitude（m） | Longitude | Latitude | Mapping rate | Mean Coverage |
| XLM2 | Xinglongmen | 52 | E 116°40′ | N 39°90′ | 88.87% | 36.98 |
| WS1 | Wushan | 220 | E 109°87′ | N 31°07′ | 90.25% | 30.05 |
| WT1 | Wutai | 1032 | E 113°25′ | N 38°72′ | 92.32% | 31.13 |
| HeN1 | Henan | 118 | E 113°38′ | N 34°29′ | 93.21% | 30.02 |
| BTM3 | Baotianman | 1182 | E 111°92′ | N 33°49′ | 90.89% | 30.15 |
| HeN2 | Henan | 118 | E 113°38′ | N 34°29′ | 90.25% | 30.58 |
| GDS5 | Guandishan | 2448 | E 111°55′ | N 37°87′ | 88.98% | 31.25 |
| GDS19 | Guandishan | 2448 | E 111°55′ | N 37°87′ | 89.58% | 32.01 |
| GDS27 | Guandishan | 2448 | E 111°55′ | N 37°87′ | 90.58% | 33.25 |
| GDS8 | Guandishan | 2448 | E 111°55′ | N 37°87′ | 91.25% | 34.25 |
| GDS1 | Guandishan | 2448 | E 111°55′ | N 37°87′ | 88.97% | 32.45 |
| WN4 | Weining | 2173 | E 104°27′ | N 26°85′ | 90.78% | 30.14 |
| WN1 | Weining | 2173 | E 104°27′ | N 26°85′ | 91.05% | 32.65 |
| KM3 | Kunming | 1930 | E 102°83′ | N 24°88′ | 89.12% | 30.13 |
| KM1 | Kunming | 1930 | E 102°83′ | N 24°88′ | 89.98% | 31.25 |
| KMC1 | Kunming | 1930 | E 102°83′ | N 24°88′ | 90.25% | 32.15 |
| KMC2 | Kunming | 1930 | E 102°83′ | N 24°88′ | 92.24% | 33.01 |
| JC6 | Jianchuan | 2199 | E 99°90′ | N 26°53′ | 90.25% | 32.05 |
| Jbg3 | Jiaobangou | 1909 | E 102°10′ | N 27°41′ | 90.14% | 30.05 |
| Jbg2 | Jiaobangou | 1909 | E 102°10′ | N 27°41′ | 89.02% | 35.23 |
| dxp4 | Daxiangping | 2264 | E 102°31′ | N 27°52′ | 90.04% | 30.01 |
| dxp2 | Daxiangping | 2264 | E 102°31′ | N 27°52′ | 88.98% | 35.21 |
| DL3 | Dali | 2157 | E 100°15′ | N 25°67′ | 89.87% | 32.01 |
| DL2 | Dali | 2157 | E 100°15′ | N 25°67′ | 90.25% | 33.05 |
| BJY1 | Bijie | 1490 | E 105°29′ | N 27°28′ | 90.14% | 30.25 |
| BJY8 | Bijie | 1490 | E 105°29′ | N 27°28′ | 89.98% | 33.25 |
| LJ1 | Lijiang | 2384 | E 100°22′ | N 26°85′ | 90.87% | 32.05 |
| LJ2 | Lijiang | 2384 | E 100°22′ | N 26°85′ | 90.25% | 33.05 |
| LJ3 | Lijiang | 2384 | E 100°22′ | N 26°85′ | 89.97% | 31.04 |
| LJ4 | Lijiang | 2384 | E 100°22′ | N 26°85′ | 90.25% | 33.05 |
| LJ5 | Lijiang | 2384 | E 100°22′ | N 26°85′ | 89.96% | 35.25 |
| LJ6 | Lijiang | 2384 | E 100°22′ | N 26°85′ | 90.04% | 36.02 |
| LJ7 | Lijiang | 2384 | E 100°22′ | N 26°85′ | 89.96% | 32.05 |
| LJ8 | Lijiang | 2384 | E 100°22′ | N 26°85′ | 90.58% | 36.05 |
| LJ9 | Lijiang | 2384 | E 100°22′ | N 26°85′ | 91.18% | 36.08 |

Table S2. Relative likelihood of the different models

| **Model** | **Max(log10(Lhoodi)^a^** | **AIC_i_^b^** | **Δi^b^** | **Model normalized**  **relative likelihood (w_i_)^b^** |
| --- | --- | --- | --- | --- |
| Model A1 | -766690638.3 | 353302140.2 | 4348409.527 | ~0 |
| Model A2 | -767779149.9 | 356589753.2 | 3698253.525 | ~0 |
| Model A3 | -769356470.4 | 354589522.3 | 2635452.236 | ~0 |
| Model A4 | -720729162.2 | 354965820.3 | 1523698.014 | ~0 |
| Model B1 | -739200638.82 | 355289547.4 | 2015896.025 | ~0 |
| Model B2 | -742253555.1 | 356985172.0 | 215486.026 | ~0 |
| Model B3 | -742377897.6 | 359863214.0 | 1548753.025 | ~0 |
| Model B4 | -741802181.4 | 354879632.2 | 698541.024 | ~0 |
| Model C1 | -703841628.8 | 352308124.0 | 0 | ~1 |
| Model C2 | -737524429.7 | 353021548.0 | 1478523.021 | ~0 |
| Model C3 | -737955129.5 | 358796852.0 | 4589632.027 | ~0 |
| Model C4 | -740117914.7 | 353258963.2 | 2587456.014 | ~0 |
| Model D1 | -743951293.6 | 353336542.3 | 5214852.061 | ~0 |
| Model D2 | -743705455.7 | 357823698.2 | 125487.098 | ~0 |
| Model D3 | -741996174.2 | 353358963.2 | 1253698.017 | ~0 |
| Model D4 | -740495214.920 | 356854712.0 | 2548756.369 | ~0 |
| Model F1 | -745012396.580 | 359586325.0 | 2548741.258 | ~0 |
| Model F2 | -739818631.175 | 356854120.3 | 369852.369 | ~0 |
| Model F3 | -745012596.580 | 357290215.2 | 3258745.025 | ~0 |
| Model F4 | -748012396.580 | 359856325.2 | 3258745.258 | ~0 |

Note: ^a^Based on the best likelihood among the 50 independent runs for each model .
^b^The calculation of AIC_i_, Δ_i_ and w_i_ are according to the methods shown in Excoffier et al. (2013).

Table S3. Candidate genes identified in the genomic windows with electron transport

| Genes | Starting pos. | Ending pos. | arabi-name | arabi-symbol | Description |
| --- | --- | --- | --- | --- | --- |
| Potri.001G015600 | 1118168 | 1123930 | AT3G45140.1 | ATLOX2,LOX2 | lipoxygenase 2 |
| Potri.002G025800 | 1663020 | 1665346 | AT4G31500.1 |  | photosystem II reaction center protein |
| Potri.002G129900 | 9745756 | 9749015 | AT4G33280.1 |  | photosynthetic electron transfer C |
| Potri.006G161500 | 15373279 | 15373431 | ATCG00270.1 |  | photosystem II reaction center protein D |
| Potri.006G190800 | 20556974 | 20558623 | AT2G42250.1 |  | photosystem II reaction center protein |
| Potri.012G071200 | 9591709 | 9594462 | AT1G05160.1 |  | photosystem II reaction center protein D |
| Potri.014G134300 | 10226370 | 10227093 | AT2G47880.1 |  | Glutaredoxin family protein |
| Potri.019G096400 | 12731920 | 12735690 | AT5G47530.1 |  | Auxin-responsive family protein |
| Potri.009G016500 | 2730875 | 2731420 | ATCG00280.1 |  | photosystem II reaction center protein C |
| Potri.011G074600 | 7203770 | 7204270 | ATCG00730.1 |  | photosynthetic electron transfer D |
| Potri.011G074700 | 7205250 | 7205949 | ATCG00720.1 |  | photosynthetic electron transfer B |
| Potri.011G074800 | 7206842 | 7207063 | ATCG00710.1 |  | photosystem II reaction center protein H |
| Potri.013G142700 | 14882770 | 14884113 | ATCG00140.1 |  | ATP synthase subunit C family protein |
| Potri.013G142800 | 14884552 | 14887338 | ATCG00120.1 |  | ATP synthase subunit alpha |

Table S4. Candidate genes identified in the genomic windows with apoptotic process and programmed cell death

| Genes | Starting pos. | Ending pos. | arabi-name | Description |
| --- | --- | --- | --- | --- |
| Potri.T048266 | 86193 | 91295 | AT1G69550.1 | disease resistance protein (TIR-NBS-LRR class) |
| Potri.T050200 | 252965 | 263334 | AT1G69550.1 | disease resistance protein (TIR-NBS-LRR class) |
| Potri.017G145300 | 15303000 | 15304281 | AT3G14470.1 | NB-ARC domain-containing disease resistance protein |
| Potri.017G102900 | 11966965 | 11976728 | AT5G17680.1 | disease resistance protein (TIR-NBS-LRR class), |
| Potri.017G137750 | 14629334 | 14630074 | AT3G14470.1 | NB-ARC domain-containing disease resistance protein |
| Potri.018G003200 | 219745 | 222752 | AT3G14470.1 | NB-ARC domain-containing disease resistance protein |
| Potri.T049400 | 159945 | 162323 | AT4G12010.1 | Disease resistance protein (TIR-NBS-LRR class) |
| Potri.017G137700 | 14619638 | 14623687 | AT3G14470.1 | NB-ARC domain-containing disease resistance protein |
| Potri.013G041750 | 2936225 | 2937649 | AT3G14470.1 | NB-ARC domain-containing disease resistance protein |
| Potri.T050200 | 252965 | 263334 | AT1G69550.1 | disease resistance protein (TIR-NBS-LRR class) |
| Potri.017G144300 | 15197225 | 15199834 | AT3G14470.1 | NB-ARC domain-containing disease resistance protein |
